# Peptibody Based on FGFR1-Binding Peptides From the FGF4 Sequence as a Cancer-Targeting Agent

**DOI:** 10.3389/fphar.2021.748936

**Published:** 2021-11-12

**Authors:** Karolina Jendryczko, Jakub Rzeszotko, Mateusz Adam Krzyscik, Jakub Szymczyk, Jacek Otlewski, Anna Szlachcic

**Affiliations:** Department of Protein Engineering, Faculty of Biotechnology, University of Wroclaw, Wroclaw, Poland

**Keywords:** targeting peptides, peptide Fc fusions, peptibodies, cytotoxic conjugates, targeted therapies, FGF4, FGFR (fibroblast growth factor receptor), FGFR1

## Abstract

Targeted therapies are a promising alternative to conventional chemotherapy, with an increasing number of therapeutics targeting specific molecular aberrancies in cancer cells. One of the emerging targets for directed cancer treatments is fibroblast growth factor receptors (FGFRs), which are known to be involved in the pathogenesis and progression of multiple cancer types, specially in lung, bladder, and breast cancers. Here, we are demonstrating the development of the FGFR1-targeting agent based on the interactome screening approach, based on the isolation of binding regions from ligands interacting with the receptor. The parallel analysis by FGFR1 pull-down of chymotryptic peptides coupled with MS analysis, and PepSpot analysis yielded equivalent peptide sequences from FGF4, one of the FGFR1 ligands. Three sequences served as a basis for peptibody (Fc-fusion) generation, to overcome clinical limitations of peptidic agents, and two of them showed favorable FGFR1-binding *in vitro* and FGFR1-dependent internalization into cells. To validate if developed FGFR1-targeting peptibodies can be used for drug delivery, similar to the well-established concept of antibody–drug conjugates (ADCs), peptibodyF4_1 was successfully conjugated with monomethylauristatin E (MMAE), and has shown significant and specific toxicity toward FGFR1-expressing lung cancer cell lines, with nanomolar EC_50_ values. Essentially, the development of new effective FGFR1 binders that comprise the naturally occurring FGFR-recognition peptides and Fc region ensuring high plasma stability, and long bloodstream circulation is an interesting strategy expanding targeted anticancer agents’ portfolio. Furthermore, identifying peptides effectively binding the receptor from sequences of its ligands is not limited to FGFRs and is an approach versatile enough to be a basis for a new peptide/peptibodies development strategy.

## 1 Introduction

Targeted therapies are a promising alternative to conventional chemotherapy, with an increasing number of therapeutics targeting specific molecular aberrancies in cancer cells entering the clinical trials and being approved for the pharmaceutical market. There are many growth factor receptors, and among them, FGFR (fibroblast growth factor receptor) family is known to be frequently deregulated in multiple cancer types (Haugsten et al., 2010; Ornitz and Itoh, 2015; Hoy, 2020). The FGFRs are transmembrane receptors and together with their ligands—fibroblast growth factors (FGFs)—are responsible for the regulation of many cellular processes, for example, mitosis, proliferation, and angiogenesis (Goetz and Mohammadi, 2013; Coleman et al., 2014; Ornitz and Itoh, 2015). The FGF receptors have been reported to be involved in the pathogenesis and progression of cancer, specially for lung, bladder, and breast cancers (Haugsten et al., 2010; Jain, 2013; Katoh and Nakagama, 2014; Perez-Garcia et al., 2018). The study of FGFR-targeted therapies is mostly focused on either small-molecule tyrosine kinase inhibitors (TKIs) or monoclonal antibodies (mAbs) as therapeutic agents, and only two TKI-type drugs have been approved so far (Markham, 2019; Hoy, 2020).

Receptor-binding peptides can be used as an alternative to small-molecule inhibitors or mAbs (Lau and Dunn, 2018; Hoppenz et al., 2020). Target-binding peptides are characterized by the ease of selection and synthesis, as well as potentially better penetration properties. Increasing number of new peptides are identified, thanks to the developments in high-throughput peptide screening, computational biology, and advances in selection techniques such as phage display (Saw and Song, 2019; Schwaar et al., 2019; Bozovičar and Bratkovič, 2020; Quartararo et al., 2020).

Unfortunately, disadvantages of peptides’ therapeutic applications include their poor stability in serum and fast renal clearance (Ning et al., 2019 and Hoppenz et al., 2020). One of the recently developed ways to overcome these obstacles is engineering peptides into peptibodies. The peptibodies are genetic fusions of the Fc (fragment crystallizable) region from IgG, and a peptide with certain biological properties. They are characterized by an increased apparent affinity arising from the avidity effects caused by the dimerization of Fc fragments, combined with a long plasma half-life and slower renal clearance rate due to their size (Shimamoto et al., 2012; Wu and Sun, 2014 and Cavaco et al., 2018). Peptibodies’ half-life is also increased by the Fc-neonatal receptor system, providing molecule recycling. Moreover, Fc fragment, similarly as in antibodies, can efficiently interact with Fc receptors on innate immune effector cells, stimulating them, inducing immune response and the complement-induced cytotoxicity, and improving the overall therapeutic effect (Nimmerjahn and Ravetch, 2007).

Even though their pharmacokinetic characteristics are so favorable, so far peptibodies have only been shown to inhibit cancer cells growth by a mechanism similar to that of cancer-targeting monoclonal antibodies—simply by precluding ligand–receptor interaction. Their unexplored potential also lies in their capability to act as delivery vehicles for cytotoxic drugs, similarly to intensively developed antibody–drug conjugates (ADCs). ADCs can greatly improve selective delivery of cytotoxic drugs to tumors, allowing greater efficacy of lower doses of drugs and specific destruction of cancer cells (Doronina et al., 2006; Oflazoglu et al., 2008). Analogous attachment of highly cytotoxic drug can be performed for peptibodies, but except our earlier work, there are no reports of such conjugates (Jendryczko et al., 2020).

Here, we use the interactome screening approach to find FGFR1-binding peptides. The rationale behind this approach is the isolation of the binding regions from ligands interacting with the receptor. It is not very straightforward, as such interfaces may either be very localized and involve closely located residues, or just the opposite; they rely on many contact sites dispersed in the amino acid sequence (Tahtaoui et al., 2003; Sandomenico et al., 2009; Harigua-Souiai et al., 2015; Mertinková et al., 2020). Such binding interface characterization very often involves structural analysis with the use of either X-ray crystallography or NMR studies. To allow for a larger throughput of the analysis, as even identifying the interface does not necessarily lead to the binding peptide identification, we have decided to first identify the ligands that retain receptor-binding regions even in their unfolded state, and then rely on MS analysis to identify receptor-binding peptides. Using this approach, we have succeeded in identifying two FGFR1-binding peptides from one of the FGFR1 ligands, FGF4, and reformatted them into peptibodies. For peptibodyF4_1, we were able to conjugate it effectively with cytotoxic drug, MMAE, and the conjugate showed significant and selective toxicity toward FGFR1-expressing cells, proving that binding peptides identified with this technique can be a basis for the targeted-drug delivery systems.

## 2 Materials and Methods

### 2.1 Cell Lines

CHO-S cells (Thermo Fisher Scientific) were cultured in serum-free Power-CHO medium (Lonza) supplemented with 8 mM L-glutamine (Thermo Fisher Scientific) and 1% penicillin and streptomycin mix (Biowest). The cells were subcultered every 2–3 days at a seeding density of 0.2–0.3 × 10^6^ cells·mL^−1^. The cells were grown at 37°C with 8% CO_2_ in a shaking incubator (140 rpm).

NCI-H520 (lung squamous cell carcinoma, FGFR1-positive), NCI-H1581 (lung large cell carcinoma, FGFR1-positive), and HCC95 (lung squamous cell carcinoma, FGFR1-negative) were obtained from the American Type Culture Collection (ATCC). NCI-H520 and NCI-H1581 were cultured in RPMI 1640 (Gibco) with 10% fetal bovine serum (Biowest) and 1% penicillin and streptomycin mix (Biowest); HCC95 cells were cultured in RPMI 1640 (Gibco) with 10% fetal bovine serum (Biowest), sodium bicarbonate (Gibco), and 1% penicillin and streptomycin mix (Biowest). The cancer cell lines were cultured at 37°C with 5% CO_2_.

### 2.2 Recombinant Proteins

The extracellular domain (ECD) of FGFR1 fused to the Fc domain of human IgG1 was produced as described previously by our group (Sokolowska-Wedzina et al., 2014). The Fc domain was expressed and purified in the same manner.

FGFs, namely, FGF1, FGF2, and FGF12, were expressed in *E. coli* and purified by affinity chromatography as described before (Szlachcic et al., 2016; Krzyscik et al., 2017; and Sochacka et al., 2020). FGF16 was produced in the *E. coli* (BL21) expression strain. Bacteria were grown in the LB medium with 100 µg/ml amplicilin and 0.003% chloramphenicol to OD_600_ = 0.8 at 37°C with shaking (180 rpm). Then the protein expression was induced by adding 0.1 mM IPTG (Irish Biotech GMBH), and the culture was incubated at 30°C for 16 h. Next, the bacteria were harvested by centrifugation at 6000xg, resuspended in lysis buffer (20 mM Tris-HCl, 0.5 M NaCl, 1 mM EDTA, 0.1 mM PMSF, and pH 7.4) and homogenized by sonication. The lysate was centrifuged at 15,000xg at 4°C for 45 min. The supernatant was diluted in binding buffer (20 mM Tris-HCl, 0.5 M NaCl, 1 mM EDTA, 0.1 mM PMSF, and 1 mM DTT) and loaded on sepharose–heparin resin. The column was washed with washing buffer (20 mM Tris-HCl, 0.7 M NaCl, 1 mM EDTA, 0.1 mM PMSF, and 1 mM DTT), and proteins were eluted with elution buffer (20 mM Tris-HCl, 2 M NaCl, 1 mM EDTA, 0.1 mM PMSF, and 1 mM DTT). The elution fractions containing FGF16 were dialyzed to PBS pH 7.2 at 4°C overnight. FGF6 (#554224) and FGF8 (#SRP4053) were purchased from Biocourse and Sigma Aldrich, respectively. Recombinant FGF4, FGF5, FGF7, and FGF10 were provided by Marta Minkiewicz and Martyna Sochacka from Protein Engineering Group.

### 2.3 Fluorescent Labeling Fibroblast Growth Factor Receptor 1 and Fc Domain

ECD_FGFR1-Fc and Fc domain were fluorescently labeled with HiLyte488 dye according to manufacturers’ protocol (#225402, Thermo Fisher Scientific, United States).

### 2.4 Identification of New Peptides

#### 2.4.1 Screening of Fibroblast Growth Factor Family

Following are the FGFs’ members: FGF1, FGF2, FGF4, FGF5, FGF6, FGF7, FGF8, FGF10, FGF12, and FGF16 (∼5 µg) were denatured by 10% SDS-PAGE electrophoresis and transferred on the PVDF membrane (Milipore). Next, the membrane was blocked in 2% BSA for 1 h at room temperature and incubated with FGFR1-HiLyte488 (0.1 mg/ml) diluted in 2% BSA in PBS overnight at 4°C with rotating. The signal from FGFR1 was quantified using Image Lab software. The membrane was stripped by reprobing buffer (#21059, Thermo Fisher Scientific), washed, and blocked with 2% BSA. Incubation with the Fc domain-HiLyte488 was carried out in the same manner.

#### 2.4.2 FGFR1: Pull-Down Assay

For digestion of FGF4, we used bovine chymotrypsin immobilized on Sepharose resin (BioSource). FGF4 (154 µg) was dissolved in digestion buffer (0.08 M Tris-HCl and 0.1 M Calcium chloride pH 7.8). Immobilized chymotrypsin (46.2 µl) was washed in 3 × 0.5 ml with digestion buffer followed by centrifugation after each washing (1000xg, 1 min). FGF4 was incubated with protease for 2 h at 37°C with shaking (600 rpm). Next, the sample was centrifuged to separate the resin and peptides. To evaluate the efficiency of protease digestion, the samples were subjected to 10% SDS-PAGE electrophoresis.

Recombinant proteins: ECD_FGFR1-Fc and Fc domain were immobilized on rProtein A Fast Flow resin (GE Healthcare) in an equimolar manner.

Afterward, the digested peptides were incubated with ECD_FGFR1-Fc protein and in parallel with the Fc domain for 1 h at 4°C with rotation. Flow-through was collected, and the resin was washed (0.1 M NaCl, 33 mM Na_2_HPO_4_, and 18 mM NaH_2_PO_4_) to eliminate unbounded proteins. Binding peptides were incubated with elution buffer (0.1 M sodium citrate pH 3.5) for 15 min at room temperature and eluted. As a control, we used the sample after digestion (input). Sequences of peptides were identified by the MS analysis.

#### 2.4.3 PepSpot Analysis

5 nM of each peptides (15 amino acids with 5 amino acids overlapped) from FGF4 were covalently bound to the cellulose-*β*-alanine-membrane (JPT, Berlin, Germany). The membrane was washed with methanol for 5 min with rotating and then washed with TBS-T (50 mM Tris-HCl pH 7.5, 150 mM NaCl, 0.1% Tween20) 3 times for 15 min and incubated with the blocking buffer (3% BSA in TBS-T) for 3 h at room temperature with shaking. Next, the membrane was incubated with 0.1 µg/ml Fc domain–HiLyte488 solution (in blocking buffer) overnight at 4°C with shaking. The signal from Fc was quantified using Image Lab software. The membrane was stripped by re-probing buffer (#21059, Thermo Fisher Scientific), washed, and blocked with 2% BSA. Incubation with the Fc domain-HiLyte488 was carried out in the same manner.

### 2.5 Expression and Purification of Peptide-Fc Fusions

Identified peptides’ sequences from FGF4 were cloned into the pLEV113 vector containing the sequence of Fc domain and expressed in CHO-S cells (Chinese Hamster Ovary). The production and purification of peptibodyF4_1, peptibodyF4_2, and peptibodyF4_3 were based on the protocol described previously for peptibodyF2 and FGFR1-Fc fusion protein (Sokolowska-Wedzina et al., 2014and Jendryczko et al., 2020) with a production time of 10 days for peptibodyF4_1 and 7 days for peptibodyF4_2 and peptibodyF4_3. Production of new peptibodies during the time was visualized by Western blotting using antihuman IgG (Fc) antibody conjugated with HRP (#ab97225) from Abcam.

### 2.6 Surface Plasmon Resonance Measurements

The interaction measurements were performed using a Biacore 3000 instrument (GE Healthcare) at 25°C in PBS with 0.05% Tween20, 0.1% BSA, 0.02% NaN_3_, and pH 7.4. The extracellular domains of FGFR1 in Fc fusions (in 10 mM sodium acetate, pH 5.0) were immobilized on the CM5 sensor chip surface (GE Healthcare) at 8800 RU using an amine coupling protocol. To determine kinetic constants of the interaction between peptibodies F4_1 or F4_2 or F4_3 and FGFR1, a set of dilutions of peptibodies at concentrations ranging from 0.15 to 4.8 μM or FGF4 (concentration from 75 to 600 nM) were injected at a flow of 30 μl/min. The association and disassociation were monitored for 180 and 240 s, respectively. Between injections, 10 mM glycine (pH 1.5) was applied to regenerate the sensor chip surface. The data were analyzed using the BIAevaluation 4.1 software (GE Healthcare). SPR measurements showed that the peptibodies did not interact 1:1 with the receptor, and therefore, the data did not fit to the standard 1:1 Langmuir binding model. For this reason, we used steady-state affinity analysis (which is particularly suitable for measurements of weaker interactions) to determine the K_D_ value without computing kon and koff (Suh et al., 2014). Response values from the last 10 s of the association phase were averaged and used to determine the K_D_.

### 2.7 Fluorescence Microscopy

Experiments were performed on HCC95 and NCI-H520 cell lines (10^4^ cells per well). Before experiments, cells seeded on 96-well plates were starved for 4 h.

The cells were incubated with peptibodyF4_1 (4 µg per well) or peptibodyF4_3 (4 µg per well), and after 10- and 30-min, plates were cooled to stop internalization. The cells were washed with PBS and fixed with 4% paraformaldehyde for 15 min at room temperature, permeabilized in 0.3% Triton X-100 at 4°C for 10 min, and blocked with 2% BSA and 0.3 M glycine in PBS-T for 1 h at room temperature. The cells were incubated overnight at 4°C with primary antibodies: rabbit anti-EEA1 antibody (#2411S Cell Signalling), rabbit anti-LAMP1 antibody (#ab24170, Abcam), or anti-FGFR1 antibody (#3472 Cell Signaling), diluted in PBS-T with 2% BSA, followed by further 1 h incubation with Donkey anti-Rabbit (H + L) ReadyProbes secondary antibody, Alexa Fluor 594 (#R37119, Thermo Fisher Scientific), diluted in 2% BSA in PBS-T. Peptibodies were labeled with Zenon™ Human IgG Labeling Kit (#Z25402, Invitrogen, Thermo Fisher Scientific) for 1 h at room temperature and fixed with 4% paraformaldehyde for 15 min at room temperature. Nuclei were labeled with NucBlue reagent (#R37605, Thermo Fisher Scientific).

Wide-field fluorescence microscopy was performed with a Zeiss Axio Observer Z1 fluorescence microscope using a LD-Plan-Neofluar 40/0.6 objective and Axiocam 503 (Zeiss, Germany). Images were processed with Zeiss ZEN 2.3 software (Zeiss, Germany) and Adobe Photoshop CS6 (Adobe, San Jose, CA, United States).

### 2.8 Flow Cytometry Analysis

Serum-starved NCI-H520 cells were incubated with increasing concentrations (1, 10, 100, and 1,000 nM) of DyLite650-labeled peptibodyF4_1, labeled according to manufacturers’ protocol, and incubated for 30 min at 37°C. In order to stop the receptor trafficking, cells were placed on ice and washed with ice-cold PBS. Non-internalized peptibody was removed from the cell surface by washing in acid stripping buffer. The cells were detached with 10 mM EDTA-PBS and washed in FACS buffer. Flow cytometric analysis was performed using a NovoCyte 2060R instrument and NovoExpress software (ACEA Biosciences, San Diego, CA, United States).

### 2.9 Conjugation with Monomethylauristatin E

#### 2.9.1 Optimization of Conjugation Reaction Conditions

Optimization conditions of conjugation of peptibodyF4_1 included composition of reaction buffer (PBS pH 7.2 or PBS pH 7.2 with 1 M urea and 5% glycerol), different excess concentrations of vcMMAE (5x, 10x, 15x, and 20x) over protein, and concentration of peptibodyF4_1 used in the reaction of conjugation (0.1 mg/ml—1 mg/ml). Reduced protein was incubated with cytotoxic drug for 1 h and left overnight at 15°C. For peptibodyF4_3, we verified the optimal composition of reaction buffer (PBS pH 6.5 or PBS pH 6.5 with 1 M urea, 5% glycerol, and 1 mM EDTA) under different conditions of reduction of disulfide bonds within the hinge region (1 mM TCEP RT/10xTCEP, RT/1 mM TCEP 30 min RT, 30 min 30°C, 120 min 37°C/10xTCEP 30 min RT, 30 min 30°C, and 120 min 37°C) and variants of MMAE (vcMMAE, PEG27vcMMAE, and PEG4vcMMAE) and incubated peptibodyF4_3 for 3 h at 15°C.

#### 2.9.2 Conjugation Reaction Scale-Up and Conjugate Purification

Conjugation of the peptibodyF4_1 was performed in reaction buffer (PBS pH 7.2, 5% glycerol, and 1 M urea). Disulfide bonds within the hinge region of the Fc domain of peptibodyF4_1 (1 mg/ml) were reduced using tris(2-carboxyethyl) phosphine (TCEP) pH 7.0 (#646547, Merck) in ten-fold molar excess over protein and incubating for 1 h at room temperature. Then, reduced and diluted 2 times, the peptibody was added to maleimidocaproyl-Val-Cit-PABC-monomethyl auristatin E (vcMMAE) (#HY-15575, MedChem Express) in 15-fold molar excess over protein and incubated at 15°C for 3 h. The peptibodyF4_1vcMMAE was purified by ion-exchange chromatography using the HiTrap CMM Sepharose FF column (GE Healthcare). The resin was washed with washing buffer (10 mM MES, pH 7.0), and the conjugate was eluted with the elution buffer (10 mM sodium citrate, 494 mM NaCl, 6 mM KCl, 5% glycerol, 0.1% PEG 3350, and pH 5.6). The purity of conjugate was confirmed by SDS-PAGE.

Conjugation of the peptibodyF4_3 was performed in reaction buffers (PBS pH 6.5 and PBS pH 6.5 with 1 M urea, 5% glycerol, and 1 mM EDTA). Reduction of disulfide bonds within the hinge region was perfomed using tris(2-carboxyethyl) phosphine (TCEP) pH 7.0 (#646547, Merck) in ten-fold molar excess over protein and incubating for 1 h at room temperature. Then peptibodyF4_3 was added to maleimidocaproyl-Val-Cit-PABC-monomethyl auristatin E (vcMMAE) (#HY-15575, MedChem Express) in 10- or 15-fold molar excess over protein (PBS/PBS with 1 M urea, 5% glycerol, and 1 mM EDTA) and incubated at 15°C for 3 h. Due to visible precipitation, conjugation reactions were centrifuged at 15000xg for 20 min; supernatants were loaded on the ProteinA-Sepharose column (MabSelect Sure, GE Healthcare) and eluted with 0.1 M citric acid pH 3.0.

The drug-to-protein ratio (DPR) was determined spectrophotometrically (Chen and Ducry, 2013). Absorbance of peptibodyF4_1-vcMMAE in PBS was measured at 248 and 280 nm, and extinction coefficients for MMAE (*ε*_MMAE_^248^ = 15 900 L/mol cm^−1^ and *ε*_MMAE_^280^ = 1 500 L/mol cm^−1^) and peptibodyF4_1 (*ε*_pep_^248^ = 20 166 L/mol cm^−1^ and *ε*_pep_^280^ = 46 786 L/mol cm^−1^) were used.

### 2.10 Conjugate Cytotoxicity Assessment

The cytotoxicity of peptibodyF4_1vcMMAE was performed on FGFR1-negative cell line (HCC95) and FGFR1-positive cell lines (NCI-H520 and NCI-H1581). Cells were seeded on a 96-well plate (5,000 cells per well) and incubated for 24 h at 37°C with 5% CO2. Conjugate was added to the cells in different concentrations (from 0.05 to 200 nM) and incubated for 96 h. The cytotoxicity of peptibodyF4_1vcMMAE was determined by Alamar Blue reagent (Invitrogen) according to the manufacturer’s protocol. The fluorescence intensity with an excitation of 560 nm and an emission of 590 nm was measured using an Infinite M1000 PRO plate reader (Tecan). Every experiment was performed in triplicates. EC_50_ values were calculated based on the Hill equation using Origin 7 software (Northampton, MA).

## 3 Results

### 3.1 Identification of New Fibroblast Growth Factor Receptor 1–Binding Peptides

**FIGURE 1:**
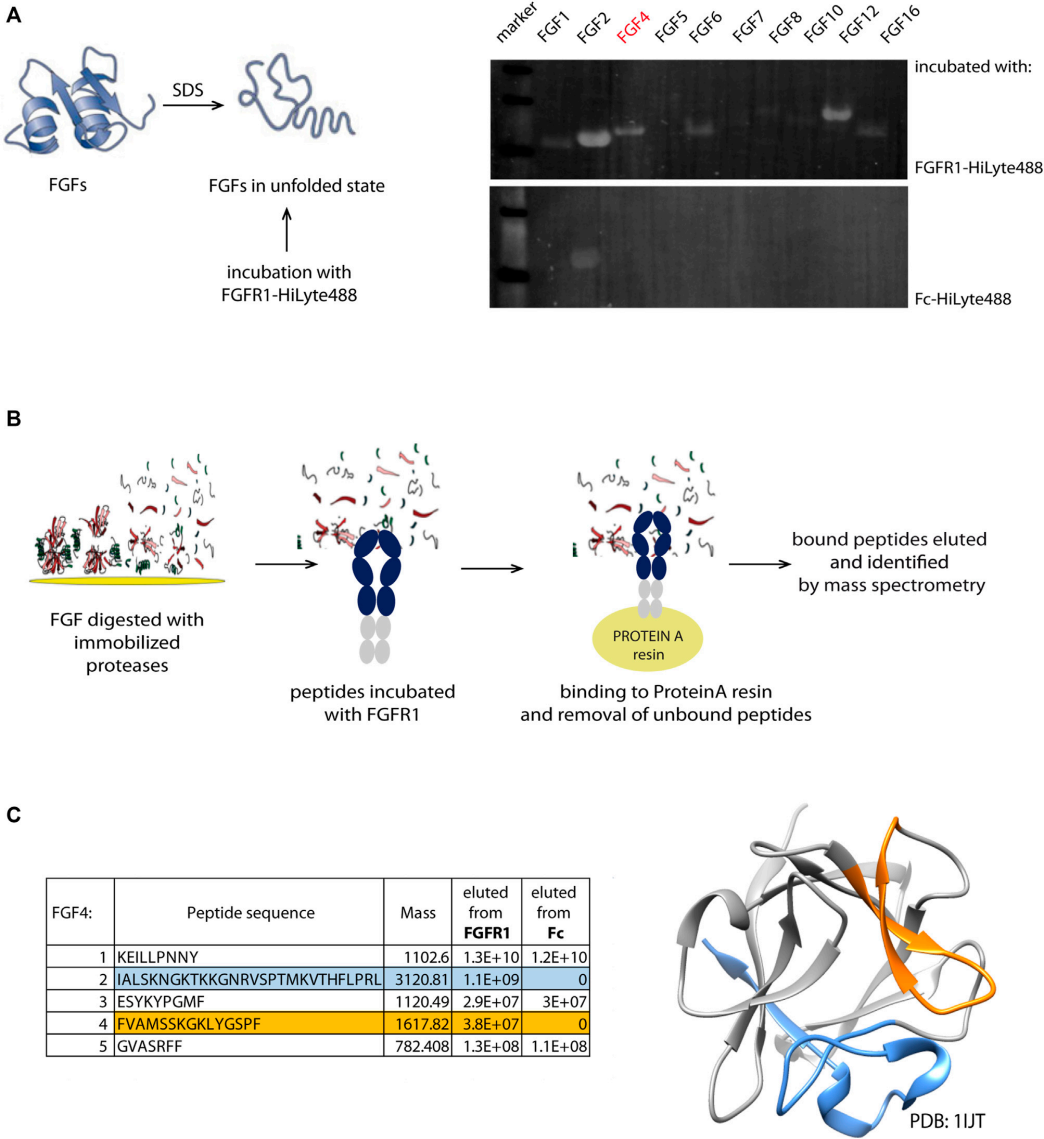
Identification scheme of new linear FGFR1-binders. **(A)** Members from FGF family were denatured and immobilized on the PVDF membrane. After incubation with fluorescently labeled recombinant FGFR1-Fc and Fc domain, the intensity of fluorescence signal was measured. **(B)** Schematic workflow of pull-down and mass spectrometry based identification of new FGFR1 binders from the FGF4 sequence. **(C)** Sequences of new binders (present in the eluate from immobilized FGFR1-Fc, but not Fc) identified by MS analysis marked in blue and orange in the table and on the FGF4 structure (PDB: 1IJT).

Interactome screening is one of the approaches for the identification of novel target-binding peptide sequences. For FGFR1, we chose its natural ligands—the family of fibroblast growth factors (FGFs). Identifying the FGF family members that display the ability to be bound by FGFR1 even in the unfolded state is the starting point to find the binding region and identify the linear peptide sequence. In many cases, binding sites in the proteins are scattered through their sequence and fully formed only in the properly folded protein. Therefore, our first step involves denaturing FGFs and incubating them with fluorescently labeled FGFR1—for the initial screening to verify if FGFs in their unfolded state can still bind to FGFR1 (Figure 1A). In the screening, we used a subset of FGFR1-binding proteins that are independent of co-receptors (e.g., *β*-Klotho), considering all FGFs’ subfamilies.

FGFs in the unfolded state immobilized on a PVDF membrane were incubated with fluorescently labeled FGFR1—recombinant protein composed of FGFR1 extracellular domain (FGFR1-ECD) fused with Fc (fragment crystallizable region of IgG1). Since we cannot fully control if the proteins bound to the membrane remain in the denatured state, to minimize the chance of refolding on the membrane, the assay involved fluorescently labeled FGFR1 for probing, and not primary and secondary antibodies, eliminating additional steps. We have used the PVDF membrane that binds proteins through hydrophobic interactions, which disrupted usually, making it impossible for the protein to properly form its hydrophobic core. To avoid detection of Fc-mediated binding to unfolded FGFs, the Fc alone was used as a negative control (Figure 1B). We have observed fluorescent signal corresponding to FGFR1 binding for FGF2, FGF4, FGF6, and FGF12. FGFs with highest signals from FGFR1 binding are FGF2, FGF12, and FGF4, successively. We eliminated FGF2 because of a strong signal of the Fc-domain binding, and we did not succeed in the identification of any peptide binders after proteolytic cleavage of FGF12. Here, we describe a process of FGFR1-binding site identification within FGF4 sequence, which in principle can be applied to other FGFs also.

To generate shorter peptidic fragments, we digested FGF4 with trypsin or chymotrypsin, but chymotryptic fragments were more appropriate; generated fragments were more uniform in length than tryptic peptides. Trypsin cleavage sites were clustered in few regions, yielding very short (2-5 amino acid) peptides; hence, we used chymotrypsin for further study. We used chymotrypsin immobilized on Sepharose resin, in order to minimize protease activity and carry on to next steps of peptide isolation, and to minimize the peptide purification step after digestion, which may potentially deplete the sample. In the next step, we incubated the peptide mixtures with FGFR1 or Fc-immobilized on ProteinA-Sepharose. Again, we used Fc as a negative control to exclude peptide binding to the Fc region, instead of the extracellular FGFR1 part. Finally, we analyzed the eluted peptides by mass spectrometry for the identification of potential peptidic FGFR1 binders. We obtained two peptides present in the eluate from the column with FGFR1, and not Fc (Figure 1C). They correspond to 136–150 and 179–206 regions of the FGF4 sequence.

### 3.2 PepSpot Analysis

**FIGURE 2:**
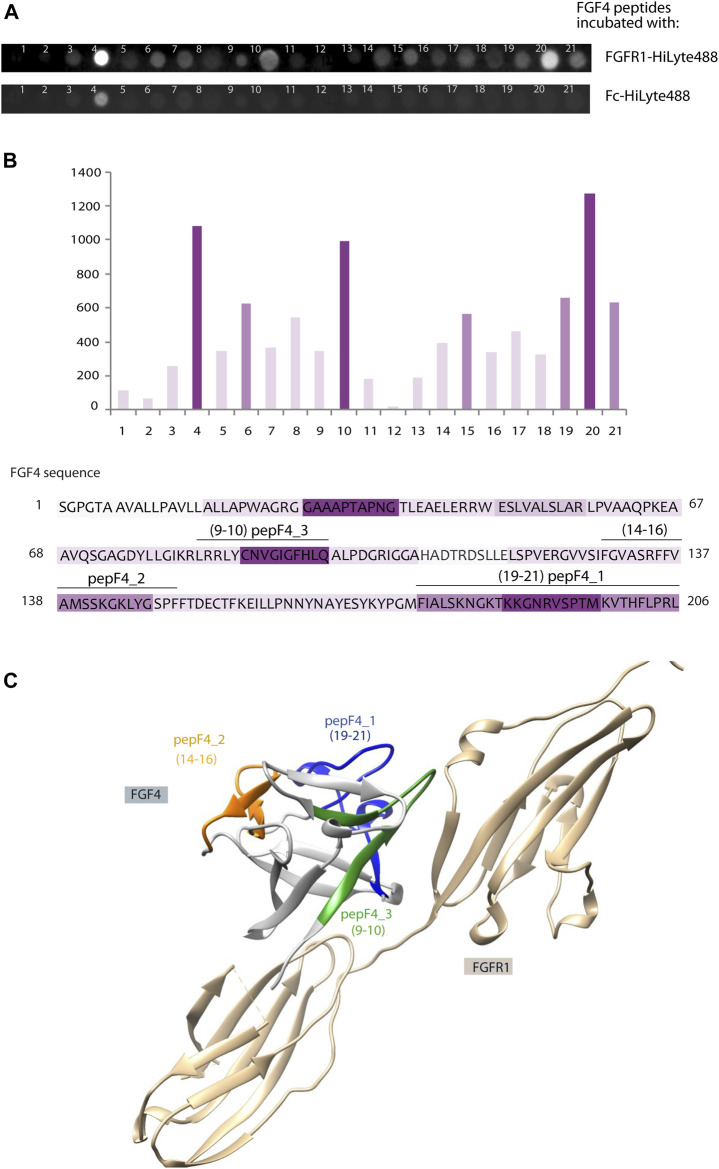
Identification of new FGFR1-binding peptides using PepSpot assay. **(A)** Membrane with immobilized peptides spanning FGF4 sequence after incubation with fluorescently labeled FGFR1 and Fc domain. **(B)** Quantitative analysis of fluorescence signals after incubation with FGFR1, performed using Fiji Software (Shindelin 2012). The signal values after incubation with Fc-HiLyte488 were subtracted from the signal after incubation with FGFR1-HiLyte488. Intensities of bars’ color correspond to the intensity of fluorescence signal. Sequence of FGF4 was colored accordingly. **(C)** Model of FGF4–FGFR1 interaction based on the structure of FGF1–FGFR1 complex (PDB: 1EVT), with peptides F4_1, F4_2, and F4_3 marked in color on the FGF4 structure.

In parallel to peptide isolation and mass spectrometry identification aforementioned, we have used a complementary approach based on peptide array immobilized on the membrane. This PepSpot customized peptide arrays consist of 15 amino-acid peptides spanning FGF4 sequence, with 10 amino acid overlap, and immobilized onto a cellulose membrane. After incubation with fluorescently labeled FGFR1 recombinant protein (extracellular domain of FGFR1 fused with Fc), we observed intense signals from peptide nos. 4, 10, 15, and 19–21. To eliminate unspecific Fc-mediated binding signal, we used a fluorescent-labeled Fc as a negative control. Incubation with Fc-HiLyte488 showed only one unspecific signal from peptide no. 4 (WAGRGGAAAPTAPNG) (Figure 2A). The fluorescence values were normalized to peptide no. 20, which had the highest fluorescence value after subtracting the Fc-derived background (Figure 2B).

Interestingly, two regions with high PepSpot scores [peptide 15 (SRFFVAMSSKGKLYG), and peptides 19–21 (KYPGMFIALSKNGKT, KNGKTKKGNRVSPTM, and RVSPTMKVTHFLPRL); Figure 2B] coincided with two regions identified with pull-down and MS approach above. Peptide no. 10 (LRRLYCNVGIGFHLQ), with positive readout in PepSpot analysis, was not detected in MS analysis. Peptide no. 4, which has shown Fc-binding, was excluded from further analysis. In order to visualize the location of these peptides within the FGF4 structure, and to check if identified peptides are positioned close to the predicted FGF–receptor interaction site, a homology model of the FGF4 molecule based on the template of FGF1–FGFR1 complex (PDB: 1EVT) was generated with the use of Swiss-Model server (Waterhouse et al., 2018) (Figure 2C). Identified peptides are not structurally clustered in one region of FGF4, and whereas peptideF4_3 is within the region predicted to be interacting with FGFR1, peptideF4_2 is located on the opposite side of the molecule. However, conformation of isolated peptides may differ from the one they adopt in a full-length folded protein, and we did not exclude peptides based on indirect structural information.

Therefore, we selected all three peptides (4_1: IALSKNGKTKKGNRVSPTMKVTHFLPRL, 4_2: FVAMSSKGKLYGSPF, and 4_3: LRRLYCNVGIGFHLQ) for further characterization in the peptibody format.

### 3.3 Peptibody Generation

**FIGURE 3:**
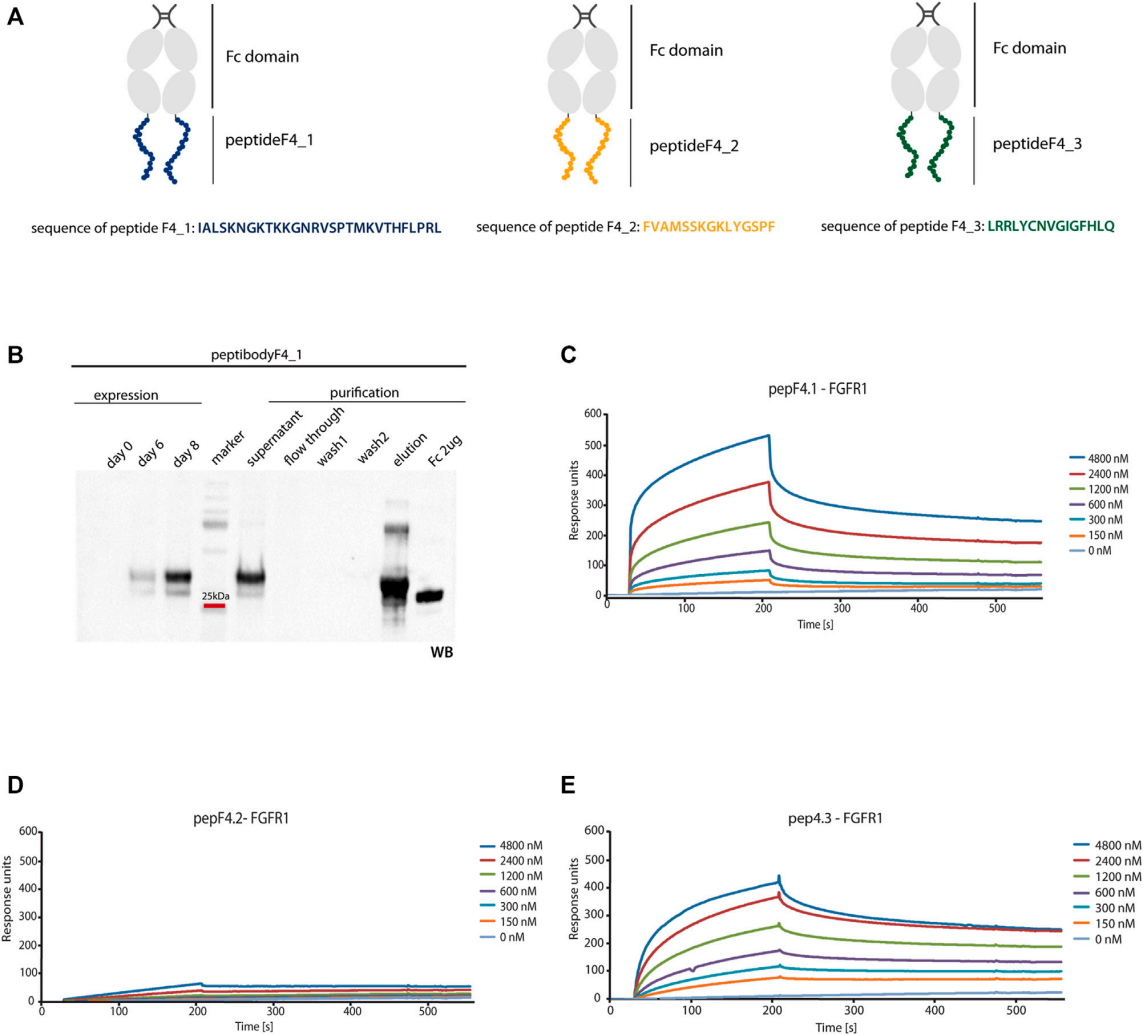
FGFR1 binding of generated peptibodies. **(A)** Scheme representing the Fc C-terminal fusion with new FGFR1-binding peptides identified from FGF4 sequence. PeptideF4_1 is marked in blue, peptideF4_2 in orange, peptideF4_3 in green, and Fc domain in gray. **(B)** Peptibodies were overexpressed in CHO-S cells and purified by affinity chromatography on ProteinA-resin. Detection of peptibody was carried out by western blotting using antibody recognizing the Fc domain. Example of expression and purification process Western blot analysis is presented for peptibodyF4_1. **(C–E)** SPR measurements determined the affinity of peptibodies to FGFR1. FGFR1 was immobilized on SPR sensors and incubated with different concentrations of peptibodyF4_1 **(C)**, peptibodyF4_2 **(D)**, and peptibodyF4_3 **(E)**. K_D_ values determined for peptibodyF4_1 and peptibodyF4_3 were 2.99 × 10^−6^ M and 1.09 × 10^−6^ M, respectively. PeptibodyF4_2 did not show measurable FGFR1 binding.

To overcome clinical limitations of peptidic agents (e.g., short life-time and fast renal clearance), we genetically fused a newly identified peptide F4_1, F4_2, and F4_3 at the C-terminus of crystalizable fragment of human IgG1 (Fc domain) and created peptibodyF4_1, peptibodyF4_2, and peptibodyF4_3, respectively. As aforementioned, sequences of peptides F4_1, F4_2, and F4_3 are derived from FGF4 (residues 179–206, 136–150, and 83–97, respectively) (Figure 3A). We introduced additional glycine residue as a spacer between the Fc domain and the targeting peptide.

We overproduced peptibodies in CHO-S cells and purified them using affinity chromatography based on the protocol previously developed in our group (Figure 3B) (Sokolowska-Wedzina et al., 2014 and Jendryczko et al., 2020). We obtained 20 mg of at least 95% pure peptibodyF4_1 and 15 mg of at least 95% pure peptibodyF4_2 from 1 L of cell culture. PeptibodyF4_3 was characterized with much lower levels of expression and yielded between 0.5 and 3 mg of 95% pure protein from 1 L of culture, depending on the expression batch. The identity and purity of peptibodies were confirmed by Coomassie Brilliant Blue staining and Western blotting.

### 3.4 Characterization of Fibroblast Growth Factor Receptor 1–Binding and Internalization Properties of Generated Peptibodies

The crucial feature of newly obtained peptibodies is their ability to efficiently bind FGFR1 and to be efficiently and specifically internalized into FGFR1-expressing cells. Therefore, new peptibodies were characterized with regard to their binding to FGFR1 with the use of SPR (surface plasmon resonance), and with fluorescence microscopy to evaluate the level of receptor binding and internalization into FGFR1-expressing cells.

For SPR analysis, recombinant FGFR1 was immobilized on the sensor, and association and dissociation profiles of newly developed peptibodies were measured. We observed binding signal for peptibodyF4_1, and titration experiment showed that the binding is dependent on the peptibody concentration (Figure 3C). PeptibodyF4_1 binds FGFR1 with K_D_ of 2.99 × 10^−6^ M, as estimated from steady-state values. For peptibodyF4_2, there were negligible signal levels, suggesting that this variant does not show FGFR1 binding (Figure 3D). PeptibodyF4_3 showed FGFR1 binding and the measured K_D_ equals to 1.09 × 10^−6^ M (Figure 3E), similar to peptibodyF4_1. Based on these results, we chose peptibodies F4_1 and F4_3 for further analysis. FGFR1 binding affinities were significantly low for FGF4, for which K_D_ equaled to 6.57 × 10^–8^ M, as measured by SPR (Supplementary Figure S1A).

To check if *in vitro* FGFR1 binding translates to efficient binding and internalization into FGFR1-expressing cells, we used lung cancer cell lines characterized with regard to FGFR1 expression levels. Internalization into NCI-H520 cells, previously characterized as lung cancer cells with increased levels of FGFR1 expression, was compared to internalization into HCC-95 lung cancer cells with low levels of FGFR1 (Wynes et al., 2014). To evaluate if micromolar affinities to FGFR1 are sufficient to observe internalization into FGFR1-positive cells, NCI-H520 cells were incubated with various concentrations of the peptibody and analyzed by flow cytometry (Supplementary Figure 1B). We can observe internalization signal for submicromolar concentrations of peptibody, suggesting that relatively low *in vitro* affinities to FGFR1, measured by SPR, still allow for effective peptibody binding to the receptors expressed in the cells.

Since peptibodyF4_1 is a candidate for a cytotoxic drug carrier, we checked by fluorescence microscopy if internalized peptibodies colocalize first with endosomal proteins (with anti-EEA-1 antibodies), and then at later stage with lysosomal markers (anti-LAMP-1 antibodies). For the release of cytotoxic drugs, the molecule is required to reach the lysosomal compartment to allow for either protein degradation or drug-linker cleavage, both leading to the release of cytotoxic cargo.

**FIGURE 4:**
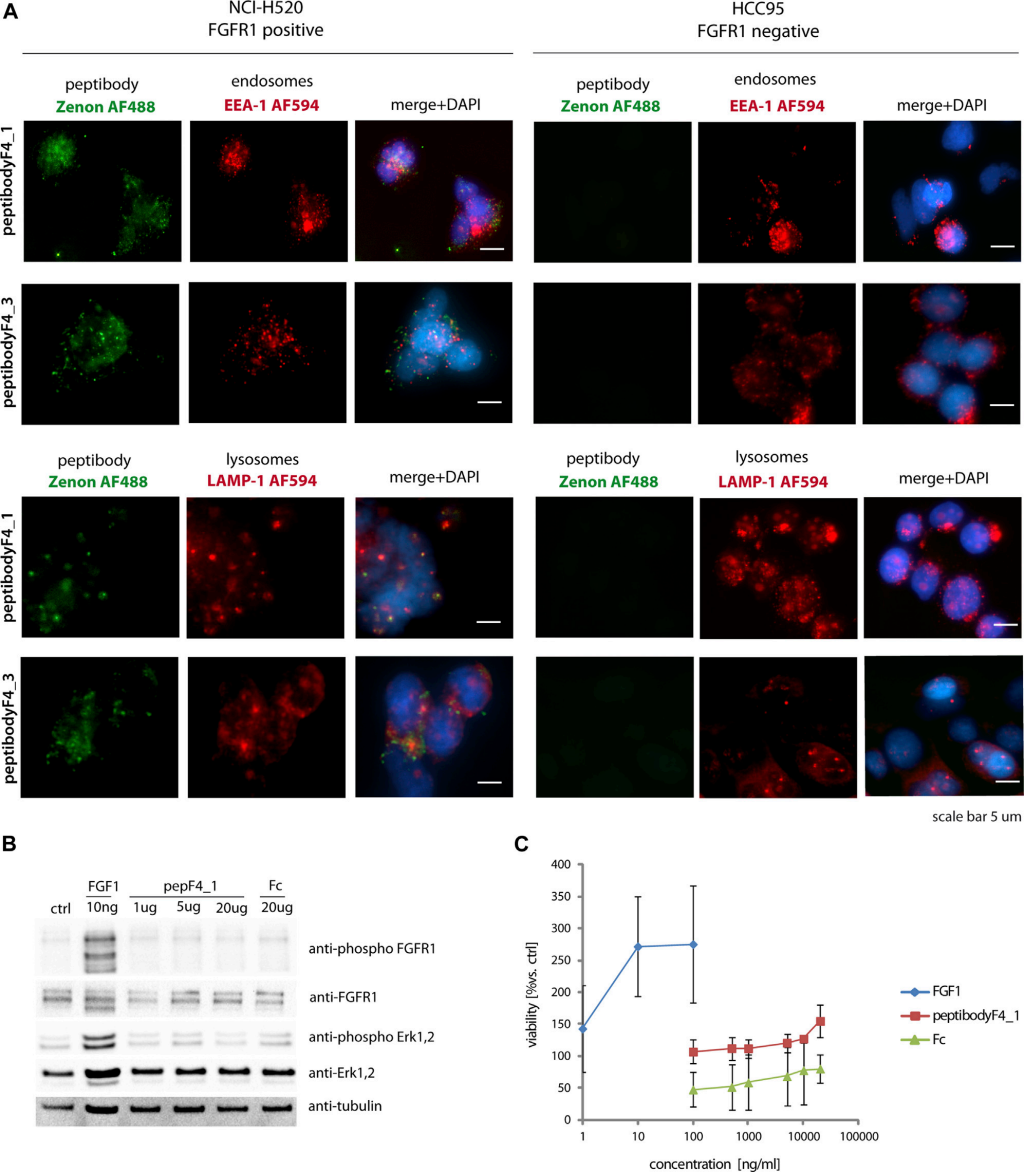
PeptibodyF4_1 internalization *via* FGFR1-dependend endocytosis. **(A)** NCI-H520 (FGFR1-positive cells) and HCC95 (control cells) were incubated with peptibodyF4_1 or peptibodyF4_3. Early endosomes were labeled with anti-EEA-1 (red), lysosomes with anti-LAMP1 (red), peptibodyF4_1 internalized was visualized with Zenon-AF488 reagent, and nuclei ware stained with NucBlue reagent. Experiments were performed in three independent replicates. **(B)** PeptibodyF4_1 binding to FGFR1 does not activate FGFR1 and downstream kinases. Activation of FGFR1 and Erk1, 2 kinases after addition of peptibodyF4_1 to NIH 3T3 fibroblasts were tested with western blot. **(C)** PeptibodyF4_1 does not cause NIH 3T3 fibroblasts proliferation after 48 h treatment. FGF1 was used as a positive control.

PeptibodyF4_1 internalization studied with fluorescence microscopy showed that it is internalized much more efficiently to FGFR1-positive NCI-H520 cells, with negligible signal observed for receptor-negative HCC-95 cells, suggesting the FGFR1-specific internalization (Figure 4A). Green fluorescence signal corresponding to peptibody labeled with Zenon488 reagent partially colocalized with red fluorescence of visualized endosomes or lysosomes, suggesting that at least a fraction of internalized peptibodyF4_1 is trafficked *via* endosomes to lysosomes. PeptibodyF4_3 behaved very similarly, as shown by fluorescence microscopy–internalized readily into FGFR1-positive cells, and not into FGFR1-negative, and showed partial colocalization with both endosomal and lysosomal markers (Figure 4B). The colocalization experiment with FGFR1 (Supplementary Figure S2A) and the fact that peptibody internalization can be competed off with unlabeled FGF4 (Supplementary Figure S2B) indicate that peptibodyF4_1 cell uptake is mediated by FGFR1.

Since binding to FGFR1 can potentially cause FGFR1 dimerization and receptor activation, we also analyzed if peptibody can induce such responses. FGFRs are activated by binding their specific ligands—FGFs. Dimerization and changes in conformation within the structure of the receptor leads to activate downstream signaling cascades (Ornitz and Itoh, 2015). While short-term stimulation leads to the activation of metabolic response, long-term response causes cell proliferation (Zinkle and Mohammadi, 2018), and any proliferative stimulation in the case of cancerous cells is highly undesirable.

FGFR1 activation was tested on mouse fibroblast cells (NIH3T3), and FGFR-dependent signaling pathway activation was used as readout of short-term response. PeptibodyF4_1 caused moderate phosphorylation of FGFR1 and activation of downstream ERK1,2 kinases compared to natural FGFR1 ligand, FGF1, and much higher concentrations of peptibody were required to see any effect on cell signaling (Figure 4B). Not surprisingly, a long-term proliferation assay on starved NIH3T3 cells stimulated with peptibodies at different concentrations showed that no significant proliferative activity can be observed upon the addition of peptibodyF4_1 to the cells (Figure 4C).

### 3.5 Conjugation of Fibroblast Growth Factor Receptor 1–Binding PeptibodyF4_1 With Cytotoxic Drug and the Analysis of Cytotoxic Potency of New Conjugates

FGFR1-binding peptibody can be used as a carrier of cytotoxic drug for its targeted delivery, analogously to antibody–drug conjugates (ADCs), well established in the clinic. The targeting molecule can specifically deliver cytotoxic compound to target-expressing cells, with negligible amounts of conjugate internalized to target-negative cells.

As a cytotoxic drug, we used monomethyl auristatin E (MMAE)—a synthetic derivative of toxin dolastatin-10. This highly cytotoxic agent with anti-mitotic properties inhibits microtubule formation (Waight et al., 2016). To ensure that the cytotoxic cargo will be released only into the cancer cells, we used valine–citrulline (vc) linker cleaved by cathepsin B in the lysosomal compartments, and well characterized in clinical studies (Bargh et al., 2019).

**FIGURE 5:**
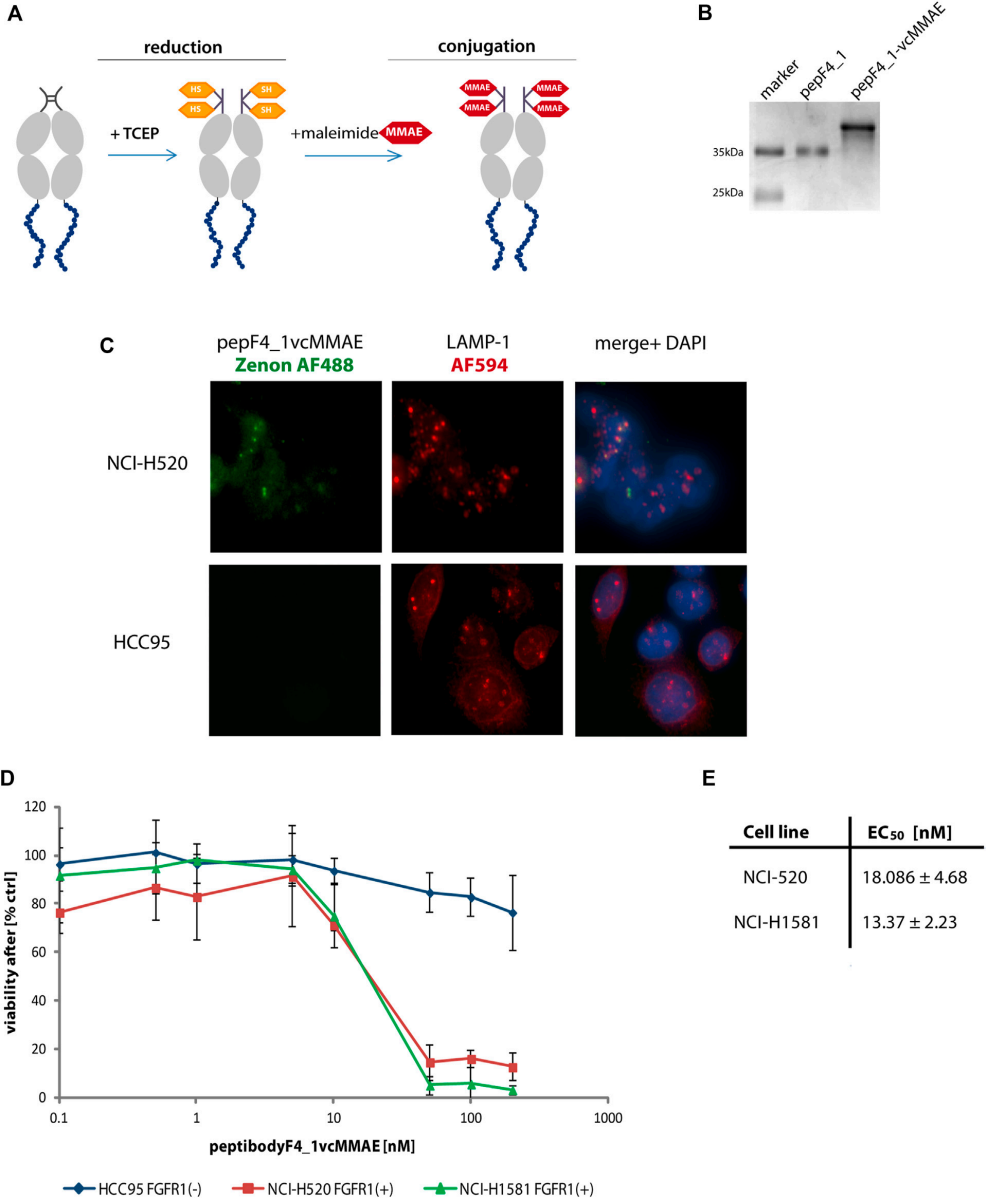
PeptibodyF4_1 was efficiently conjugated with monomethylauristatin E. **(A)** Scheme representing the stages of conjugation with cytotoxic payload *via* maleimide reaction. **(B)** Complete substitution of a peptibodyF4_1 by a monomethylauristatin E was confirmed by SDS-PAGE electrophoresis and CBB staining. **(C)** NCI-H520 (FGFR1-positive cells) and HCC95 (control cells) were incubated with peptibodyF4_1vcMMAE conjugate. Lysosomes were labeled with anti-LAMP1 antibody (red), peptibodyF4_1vcMMAE conjugate was visualized with Zenon-AF488 reagent, and nuclei ware stained with NucBlue reagent. **(D)** Cytotoxicity of peptibodyF4_1 towards cancer lung cell lines. Comparison of cytotoxic effect of conjugate on FGFR1-positive cell lines (NCI-H520, NCI-H1581) and FGFR1-negative cell lines (HCC95). All experiments were normalized to the values for non-treated cells recognized as 100%. The error bars represent ±SD for *n* = 3 experiments. **(E)** EC_50_ values for peptibodyF4_1vcMMAE for NCI-H520 and NCI-H1581 cell line, respectively. EC_50_ values were obtained based on the Hill equation using Origin 7 software (Northampton, MA).

Our conjugation strategy based on maleimide–thiol reaction using TCEP-reduced cysteine residues in the hinge region of the Fc domain, analogously to antibody modification with drugs (Figure 5A). Unfortunately, the standard protocol used for the antibodies and Fc domain–containing proteins yielded low efficiency of conjugation and precipitate formation when the reaction was scaled up. To find the optimal conditions of conjugation of peptibodyF4_1 and peptibodyF4_3, we analyzed the effect of the various levels of excess of cytotoxic drug over protein, protein concentration, and buffer composition, including additives such as urea and glycerol (Supplementary Figures S3A,B). To increase the solubility of conjugated protein, we have also verified if a PEGylated form of vcMMAE performs better. For peptibodyF4_1 conjugation yields for PEGylated MMAE were much lower than those for vcMMAE, and final conjugation protocol included 15-fold excess of vcMMAE over reduced peptibodyF4_1 and buffer containing 5% glycerol and 1 M urea. Additives in the reaction buffer prevented protein precipitation during the reaction. PeptibodyF4_1 conjugates were purified using ion exchange chromatography to remove any remaining free vcMMAE, and the resulting conjugate purity was >80% (Figure 5B). The drug-to-protein ratio was determined spectrophotometrically by analyzing the absorbance values at 248 and 280 nm, and equaled 2.1.^33^

For peptibodyF4_3, we have also observed the highest efficiency of conjugation for vcMMAE (Supplementary Figure S4A). Reactions with PEGylated MMAE, either with short PEG chains (PEG4vcMMAE) or longer PEG chains (PEG27vcMMAE), proceeded with precipitation were not clearly visible in small-scale reactions but apparent from SDS-PAGE analysis of conjugation reactions (Supplementary Figures S4B,C). Scaling up the reactions lead to substantial precipitation, and we were not able to purify sufficient amounts of conjugate to perform any cell studies (Supplementary Figure S4D). Interestingly, the conjugate gel separation pattern for vcMMAE peptibodyF4_3 conjugation reactions was slightly different from the predicted two bands for Fc cysteine modifications, with the third band present and suggesting modification of additional cysteine. PeptideF4_3 is the only one of the three analyzed containing cysteine residues within its sequence, which poses the risk of drug modification in the targeting region.

As the linker used by us is cleaved by lysosomal proteases, we have checked cellular localization of peptibodyF4_1vcMMAE conjugate in both FGFR1-positive and FGFR1-negative cells (Figure 5C). PeptibodyF4_1vcMMAE conjugate, visualized Zenon488 reagent with green fluorescence, colocalizes with LAMP-1 staining, suggesting that drug release can occur in lysosomal compartment.

Finally, we tested the peptibodyF4_1-vcMMAE conjugate for its ability to specifically deliver drug to FGFR1 positive cancer cell lines as the ultimate prerequisite for cytotoxic peptibody conjugates is their ability to cause cell toxicity dependent on the presence of molecular target, in our case, FGFR1.

We performed cytotoxicity tests on a set of non–small-cell lung carcinoma cells (NCI-H520, NCI-H1581, and HCC-95). The NCI-H520 and NCI-H1581 cells represent FGFR1-positive cell lines, whereas HCC-95 was used as a negative control. We observed a strongly decreased viability of cell lines overexpressing FGFR1 in response to increasing concentration of peptibodyF4_1vcMMAE, while HCC-95 cells were insensitive of conjugate (Figure 5D). Strong and specific cytotoxic effect is confirmed by EC50 values for peptibodyF4_1vcMMAE: 18.1 and 13.4 nM for NCI-H520 and NCI-H1581 cell lines, respectively (Figure 5E).

## 4 Discussion

Development of new compounds targeting molecular targets is one of the rapidly developing avenues of cancer research. Monoclonal antibodies (mAbs) have become the first choice and the most commonly used targeting molecules in therapeutic applications (Carter and Lazar, 2018), with many of them investigated in clinical trials and approved for the market. Even though mAbs are characterized by high binding specificity, long serum half-time, and high retention in circulation, they also have their limitations. One of them is the size of monoclonal antibodies (>150 kDa), which is a limiting factor during penetration within a solid tumor and may cause aggregation in tissues and organs (Sharkey and Goldenberg, 2006 and Scott et al., 2012). Some reports suggest limited tumor tissue penetration for mAbs with very high target affinities (Thurber et al., 2008).

On the other end of the spectrum are small organic ligands (SOLs) and peptides recognizing therapeutically relevant targets (Li et al., 2019). They are characterized by the ease of synthesis and better penetration properties, with relatively lower target binding affinities. The disadvantages of these targeting molecules are poor stability in serum and short half-life in the bloodstream. Moreover, in contrast to mAbs and peptides, which can be standardly made against nearly any protein target, generation of SOLs is more difficult and currently has been limited to a small number of targets (e.g., carbonic anhydrase IX, folate receptor, prostate-specific membrane antigen, and somatostatin receptors) (Ginj et al., 2006; Low et al., 2008; Hillier et al., 2013; Krall et al., 2014; and Cazzamalli et al., 2018).

In order to combine some of the features of antibodies and peptides, genetic fusions of the targeting peptides and the antibody Fc region have been developed (Hall et al., 2010; Shimamoto et al., 2012). Such fusions, called peptibodies, are composed of the crystalizable Fc region (from human IgG1) and peptide with binding properties to the molecular target. Affinities of peptibodies can be superior to the affinities of binding peptides used for their generation (due to avidity effects, as Fc-fusions dimerize offering two binding sites per peptibody), and because of Fc component, they exhibit favorable pharmacokinetics and slower renal clearance rate due to their size. Therefore, peptibodies take the best of antibodies and small molecule ligands, lacking some of their pitfalls (Shimamoto et al., 2012 and Cavaco et al., 2018). Nonetheless, there are still some challenges faced due to the enthropic limitations of peptides and usually the small size of epitope affinities for targets are not as high as for that of the monoclonal antibodies.

The crucial part of the peptibody is the targeting peptide. It can be identified *via* one of the available strategies used to find the target binding peptides, with phage display techniques, screening of combinatorial libraries, microarrays, and *in silico* modeling, to name just a few. Many of the molecular targets used for cancer cell identification are receptor proteins present in abundance on the surface of the cancer cell, such as the FGFR1 protein overexpressed in multiple cancer types (MURASE et al., 2014 and Perez-Garcia et al., 2018) and used as a model target in this study. Here, we explore the idea that naturally occurring binding proteins/receptor ligands possess high-affinity binding sites, and effective binding peptides can be isolated from the ligand amino-acid sequence.

The related strategy has been presented by Marasco and colleagues (Sandomenico et al., 2009), where protein fragmentation with trypsin can lead to the formation of peptidic fragments that can serve as tools in structural studies and lead to the identification of interaction antagonists. Screening performed for exemplary alpha-helical proteins yielded peptidic antagonists, but it was limited to folded proteins and assumed retained secondary structures of isolated peptides, and as authors state, this technique may not be suitable for proteins with interaction sites located within the loop regions, as they would be much more readily cleaved during limited proteolysis. In some cases, a peptide antagonist is developed after the protein–protein interaction site is identified, with the example of FGF2 antagonist, the PTX3-derived pentapeptide ARPCA (Camozzi et al., 2006; Leali et al., 2010).

In many cases, residues involved in the interaction can be scattered throughout the protein sequence and form a binding patch only when the protein is folded and adopts its tertiary structure. Therefore, the first step in our analysis was finding out if FGFR1 interacts with any of its ligands when they are denatured in a linear form. For FGF4, FGFR1 ligand that showed FGFR1 binding even in the unfolded state, we compared two methods of identifying the interacting peptides—with the use of protease digest, pull-down, and MS analysis of FGF4 fragments, or utilizing PepSpot membrane spanning FGF4 sequence.

Interestingly, these methods identified similar sets of peptides (Figures 1, 2). Peptides F4_1 and F4_2 were found by both MS and PepSpot analyses, whereas peptide F4_3 was only identified in the latter. This may be due to the fact that FGFR1 pull-down of peptides coupled with MS analysis involves more steps and poses a higher risk of failing to identify peptides present at low quantities in the sample. Moreover, depending on the particular protein, protease cleavage patterns can result in fragmentation of binding regions. This may be the possible reason for the absence of peptideF4_3 in MS analysis results—peptideF4_3 contains the predicted chymotrypsin cleavage site, and presumably, once the binding region is fragmented, the affinity is not sufficient to allow peptide isolation by FGFR1-based pull-down. SPR measurements confirmed FGFR1 binding for peptibodies F4_1 and F4_3 (based on peptides F4_1 and F4_3), but not for peptibodyF4_2. Affinities were in the micromolar range, which is not surprising for peptidic binders (Li et al., 2008; Manfè et al., 2010; Tan et al., 2018).

There is no structural information for the FGF4–FGFR1 complex, but based on the available structures of FGF4 alone and FGFR1 complexes with other members of the FGF family, we can check the location of identified peptides relative to the potential FGFR1 interacting regions of FGF4 (Figure 2C). PeptibodyF4_2 is located in an FGFR1-distant region of FGF4, the fact that may explain the lack of binding for this molecule. Although we do not assume that isolated peptides will retain the structure they adopt in folded FGF4, peptibodyF4_3 is forming a beta-loop stabilized with hydrogen bond, and both peptibodyF4_3 and F4_1 are positioned close to the predicted receptor interaction site.

The binding strength of the developed peptibodies may seem much lower than that of the target-binding mAbs, but subsequent experiments testing internalization of peptibodyF4_1 and F4_3 into lung cancer cells with high and low levels of the FGFR1 expression demonstrated that such affinity is sufficient for selective peptibody delivery into cells. Effective cytotoxicity of peptibodyF4_1 conjugates at nanomolar concentrations may result from possible accumulation of the peptibody in the cell, specially that if the peptibody shows quick association, it can be sufficient for triggering receptor/conjugate complex internalization.

Unwanted effects, such as FGFR1 activation and stimulation of cells, are on the other hand not observed, possibly since the affinity of FGF4/FGFR1 is much higher than that for the isolated peptides, in the nanomolar range (based on the displacement assays and SPR measurements) (Zimmer et al., 1993).

The proposed application of these receptor-binding peptibodies is as cytotoxic drug carriers and conjugated with monomethylauristatin E (MMAE). Although auristatin cannot be used as a free drug due to its toxicity and adverse side effects, it is successfully used in combination with antibodies (Li et al., 2020; Zhao et al., 2020; and Currier et al., 2016). Two antibody–MMAE conjugates are approved for clinical use, and many more are tested in preclinical and clinical trials (Shefet-Carasso and Benhar, 2015; Deeks, 2019). Peptibodies can be conjugated with cytotoxic drugs similar to antibodies, and we have shown before that they are effective drug carriers (Jendryczko et al., 2020). Indeed, peptibodyF4_1 conjugates tested on FGFR1-positive and negative cancer cell lines have shown FGFR-dependent toxicity, and strikingly high EC_50_ values (Figure 5D), comparable with ADCs. As shown before, FGFR1-positive lung cancer cell line is not sensitive to the Fc-MMAE conjugate, excluding that the observed cytotoxicity of peptibodyF4_1-MMAE may be a result of the Fc-receptor mediated internalization (Jendryczko et al., 2020).

Until now, there are only two FGFR-targeted drugs approved for clinical use in bile duct cancer, and both of them are small-molecule inhibitors—erdafinitib (against FGFR2-3 overexpressed in prostate cancer) (Hoy, 2020) and pemigatinib for FGFR aberrations (Markham, 2019). A few mAbs have been developed for therapeutic FGFR1-targeting peptibodies; however, they are still at the stage of clinical testing. Taking that into account, the development of new FGFR-targeting molecules that comprise the naturally occurring FGFR-recognition peptides and Fc region ensuring high plasma stability and long bloodstream circulation is an interesting strategy expanding the targeted anticancer agents’ portfolio.

More importantly, the presented approach is not limited to FGFRs and is versatile enough to be a basis for a new peptide/peptibodies development strategy.

## Data Availability

The original contributions presented in the study are included in the article/Supplementary Material; further inquiries can be directed to the corresponding author.
