# Supplementary figures and images for: Peptibody Based on FGFR1-Binding Peptides From the FGF4 Sequence as a Cancer-Targeting Agent
Source: Front Pharmacol. 2021 Nov 12;12:748936. doi: 10.3389/fphar.2021.748936 (PMC8636100; doi:10.3389/fphar.2021.748936)

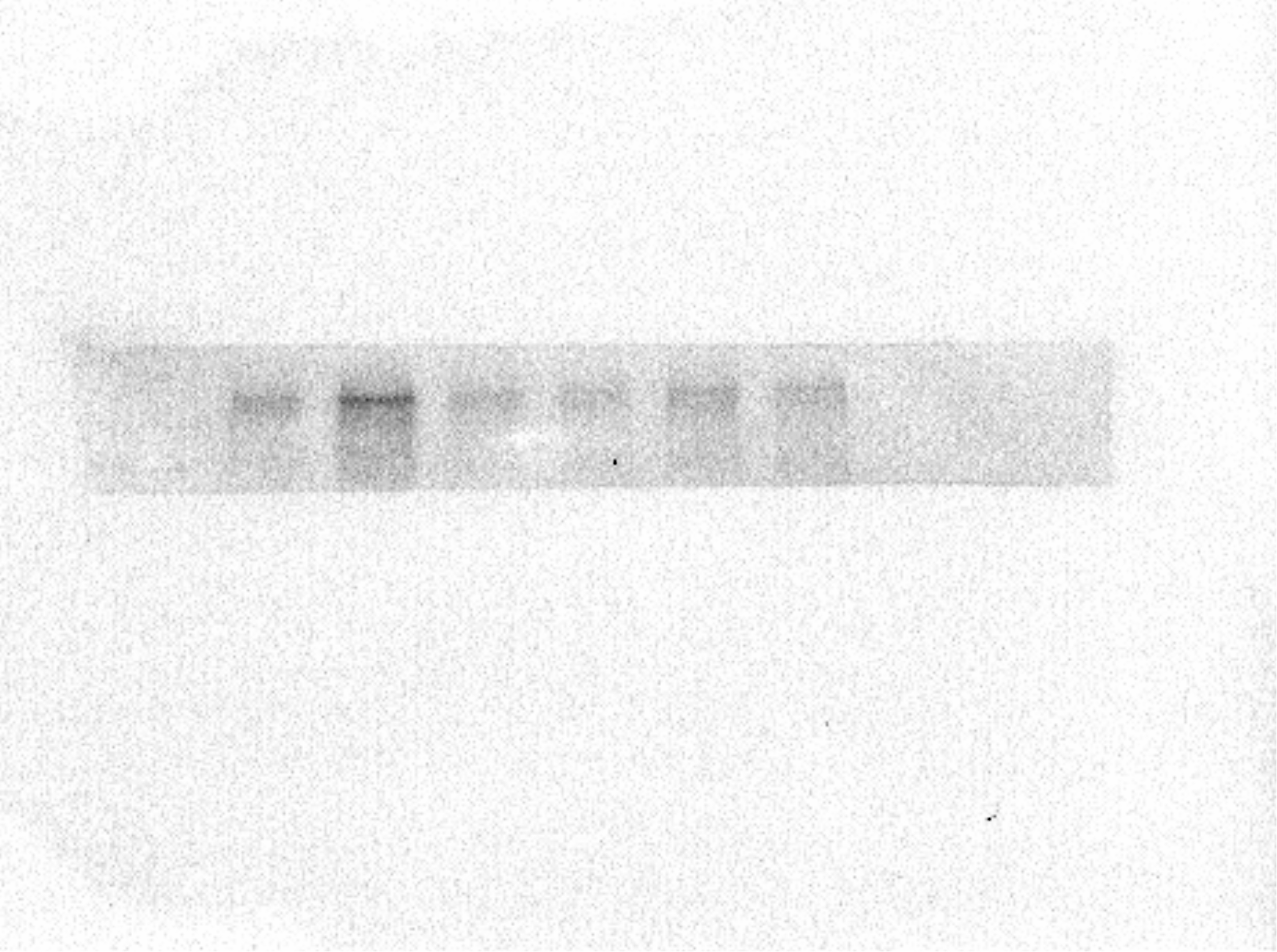

Supplement: Supplementary file 1 [file Image6.TIF]

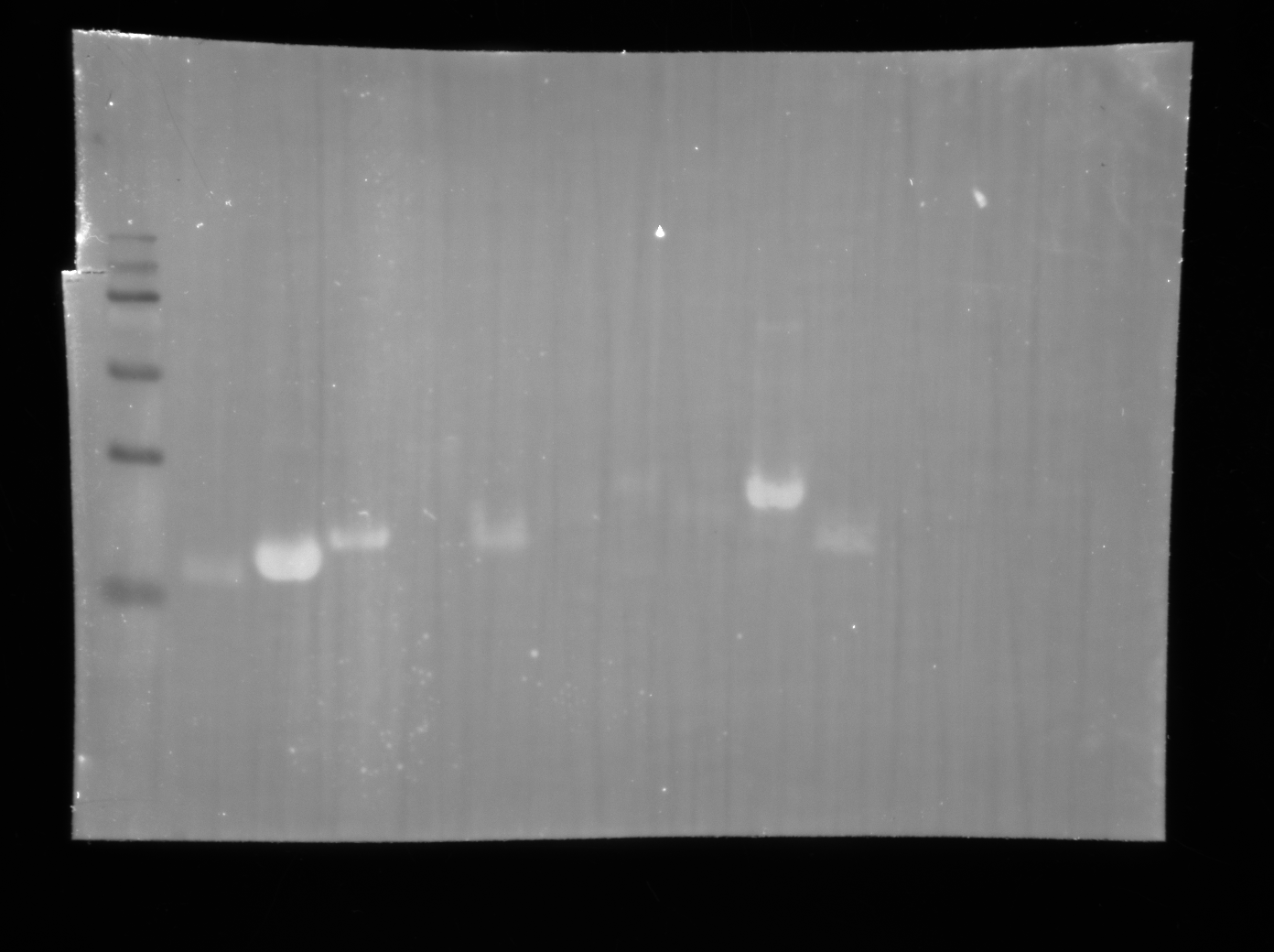

Supplement: Supplementary file 2 [file Image3.TIF]

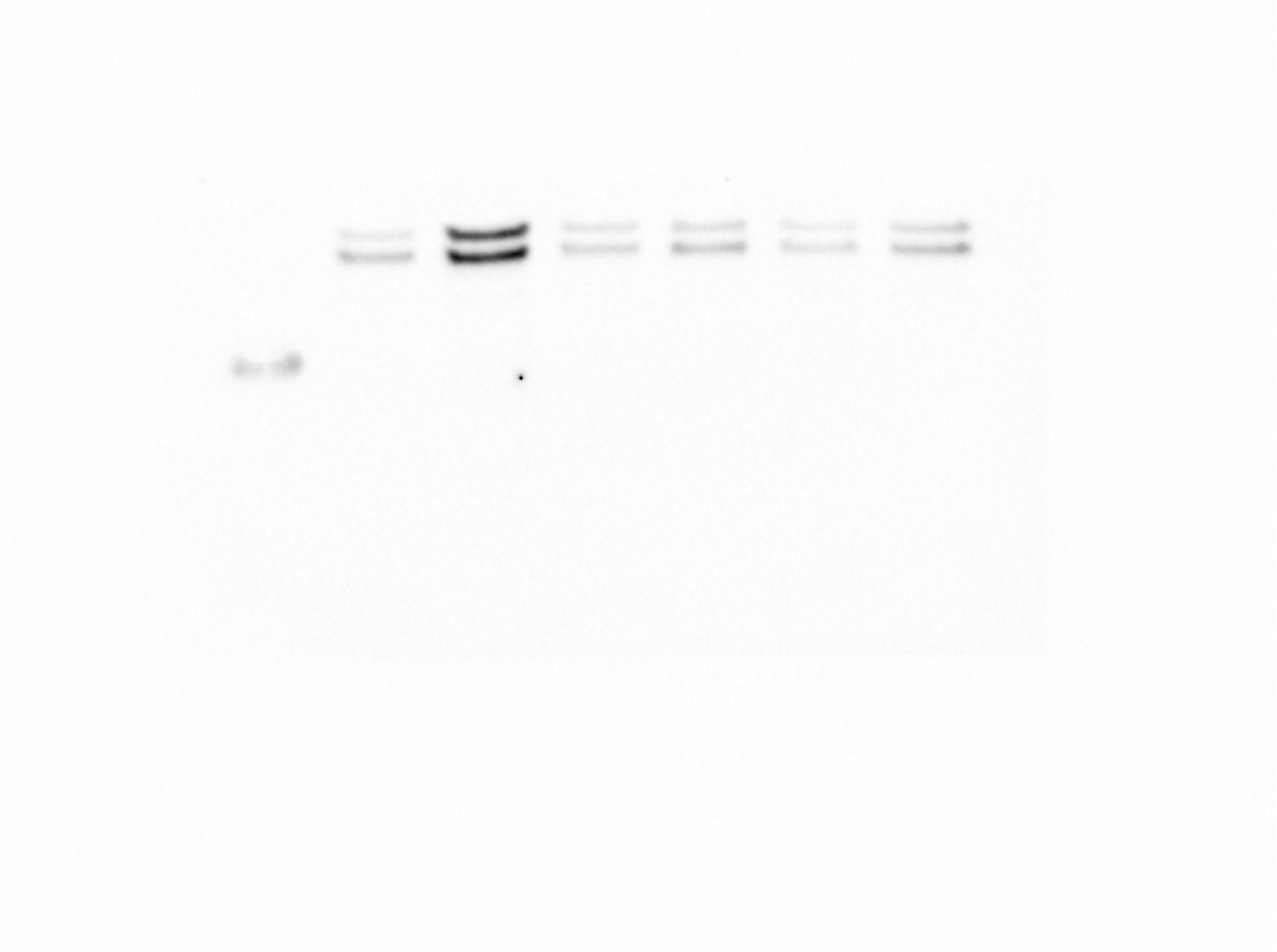

Supplement: Supplementary file 3 [file Image4.TIF]

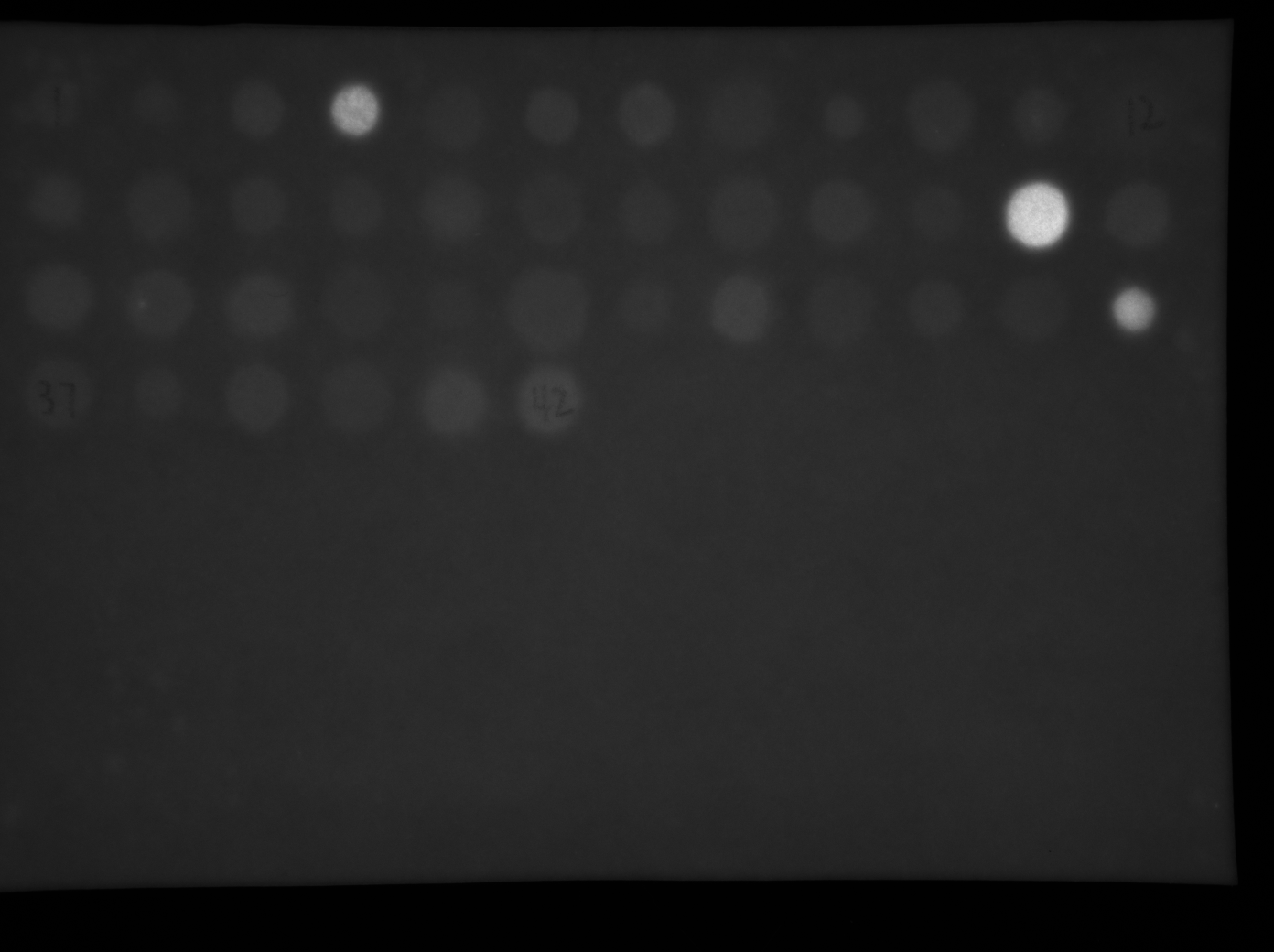

Supplement: Supplementary file 4 [file Image9.TIF]

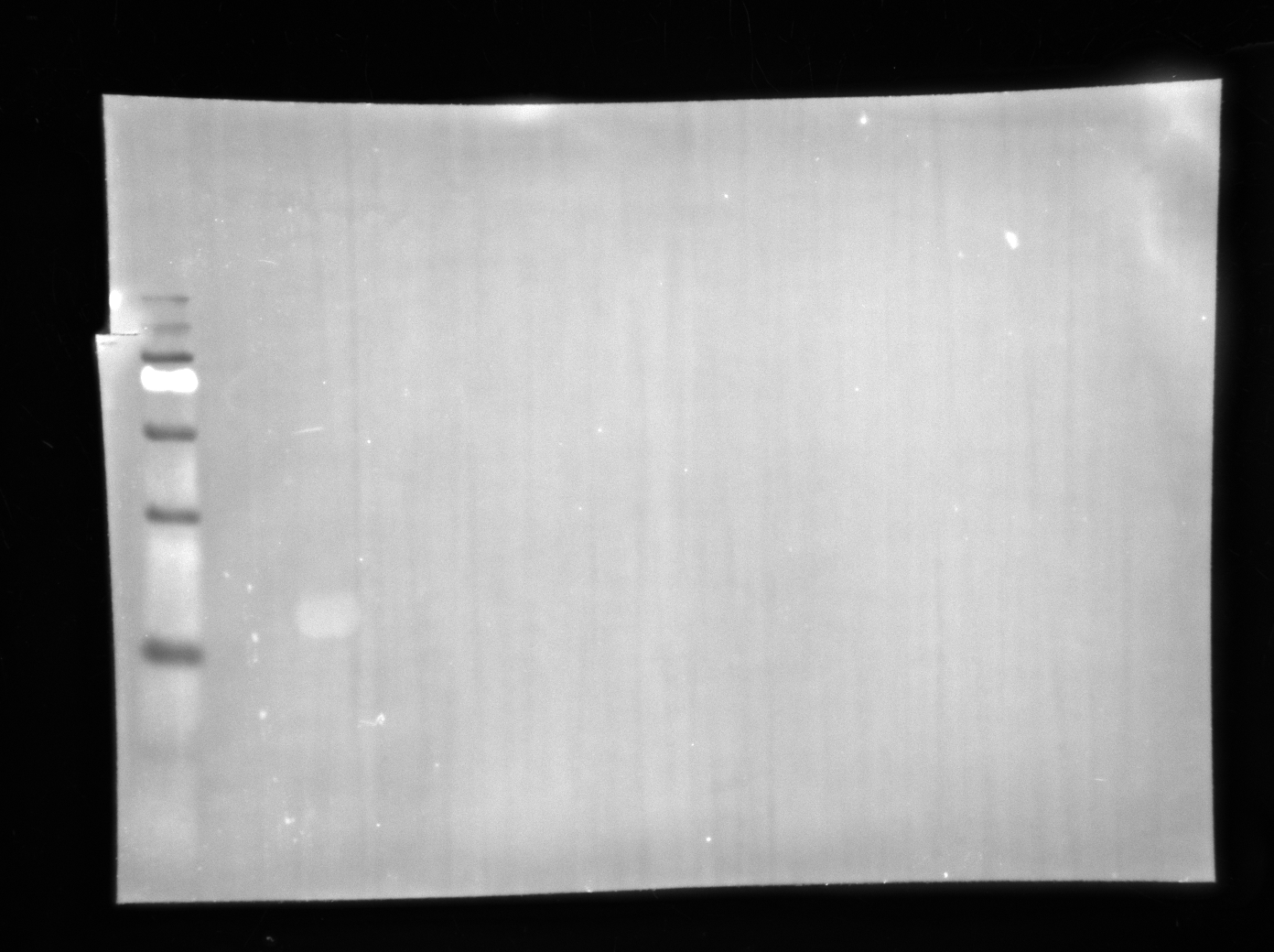

Supplement: Supplementary file 5 [file Image2.TIF]

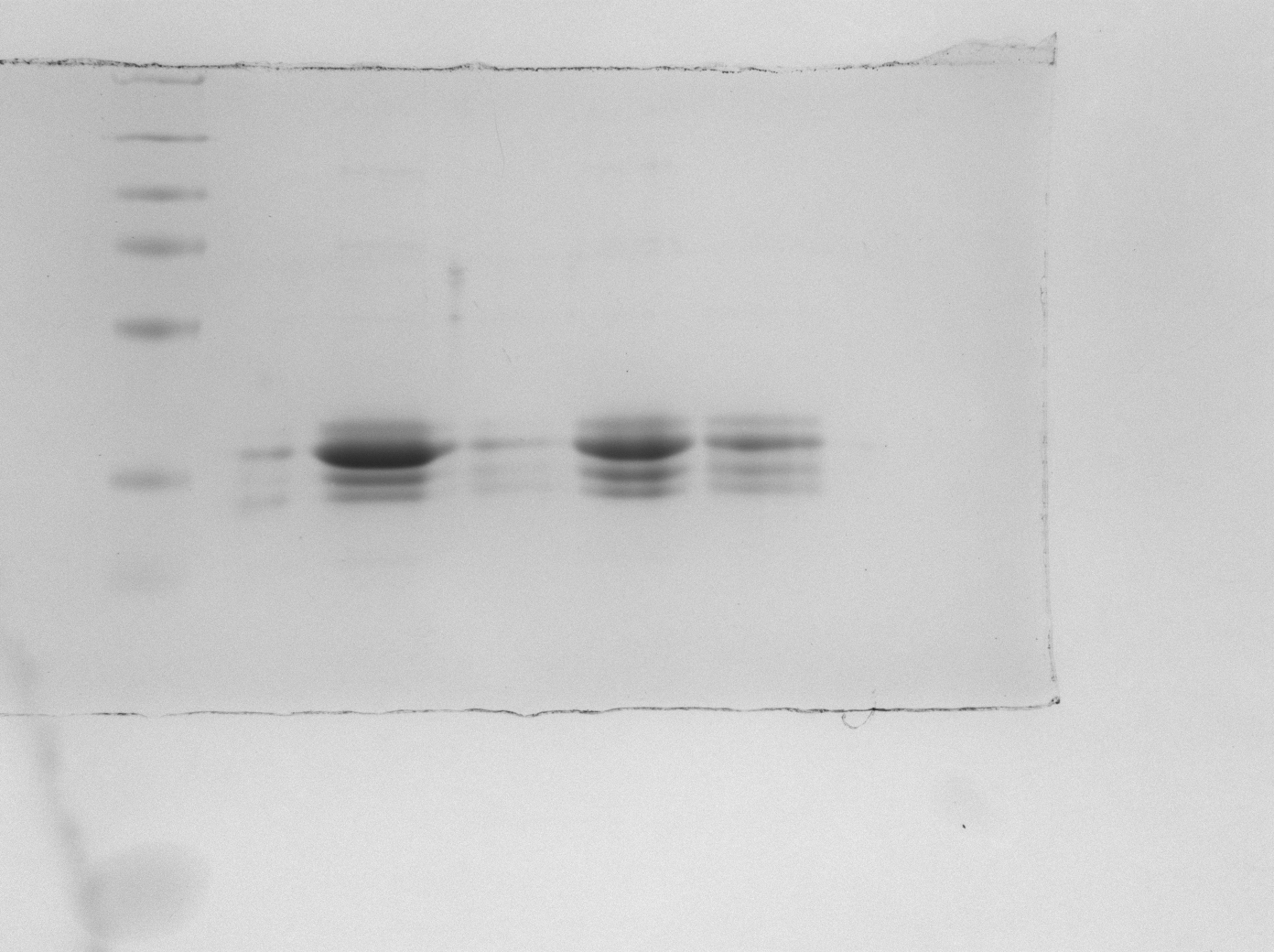

Supplement: Supplementary file 6 [file Image11.TIF]

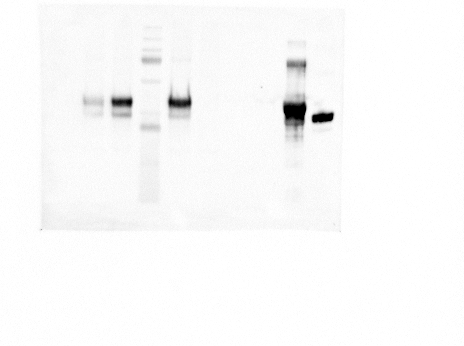

Supplement: Supplementary file 7 [file Image1.TIF]

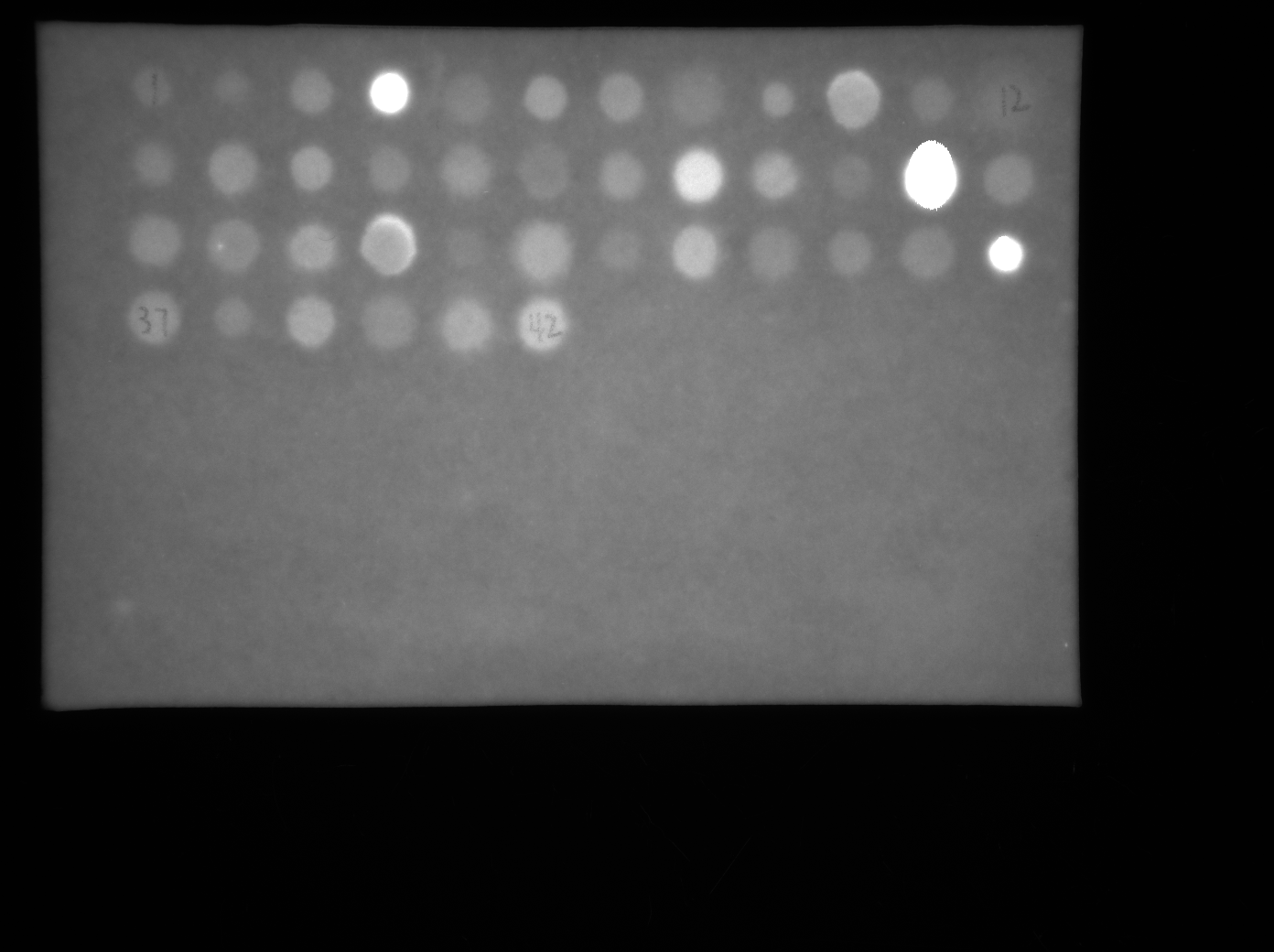

Supplement: Supplementary file 8 [file Image10.TIF]

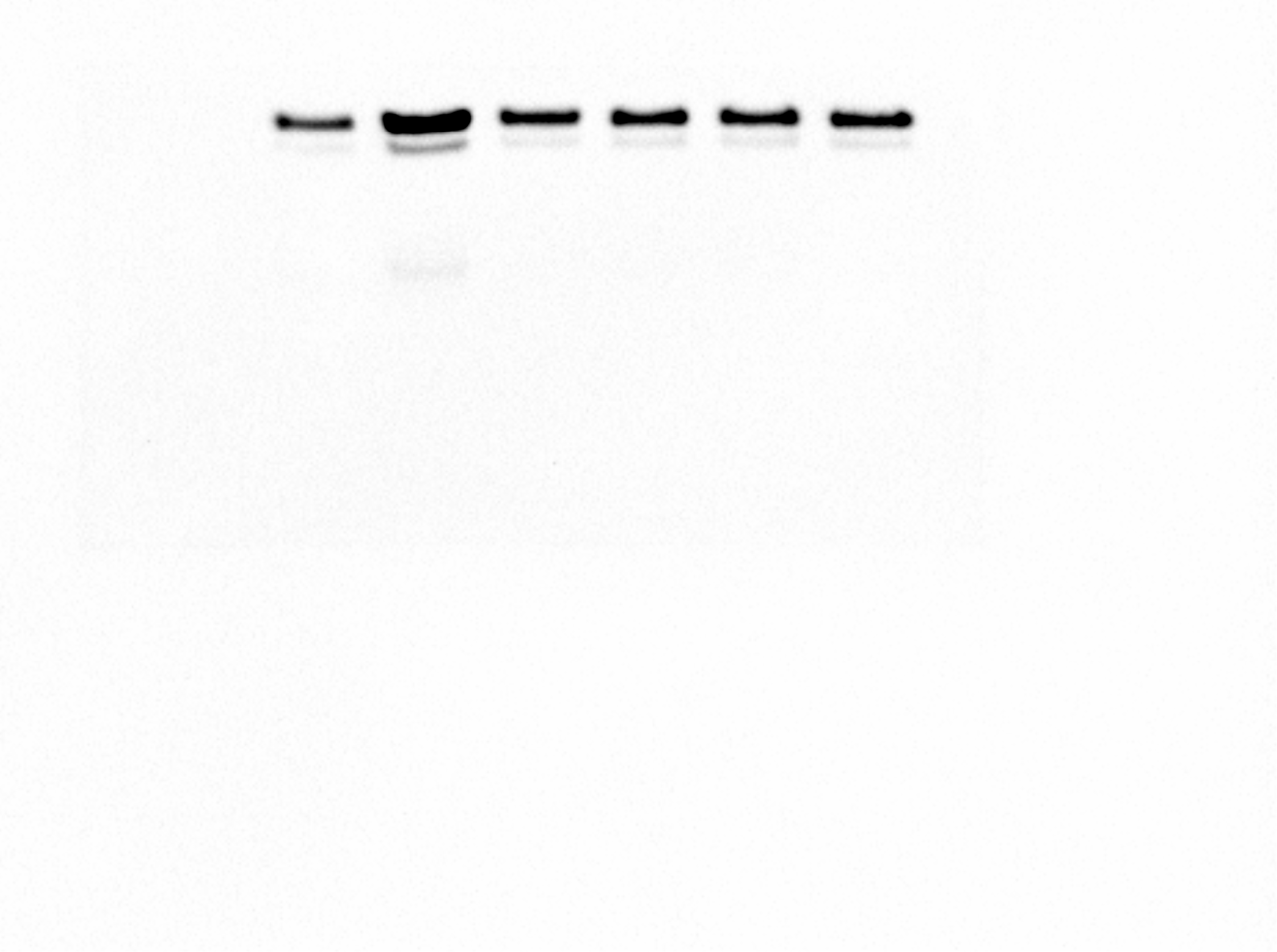

Supplement: Supplementary file 9 [file Image7.TIF]

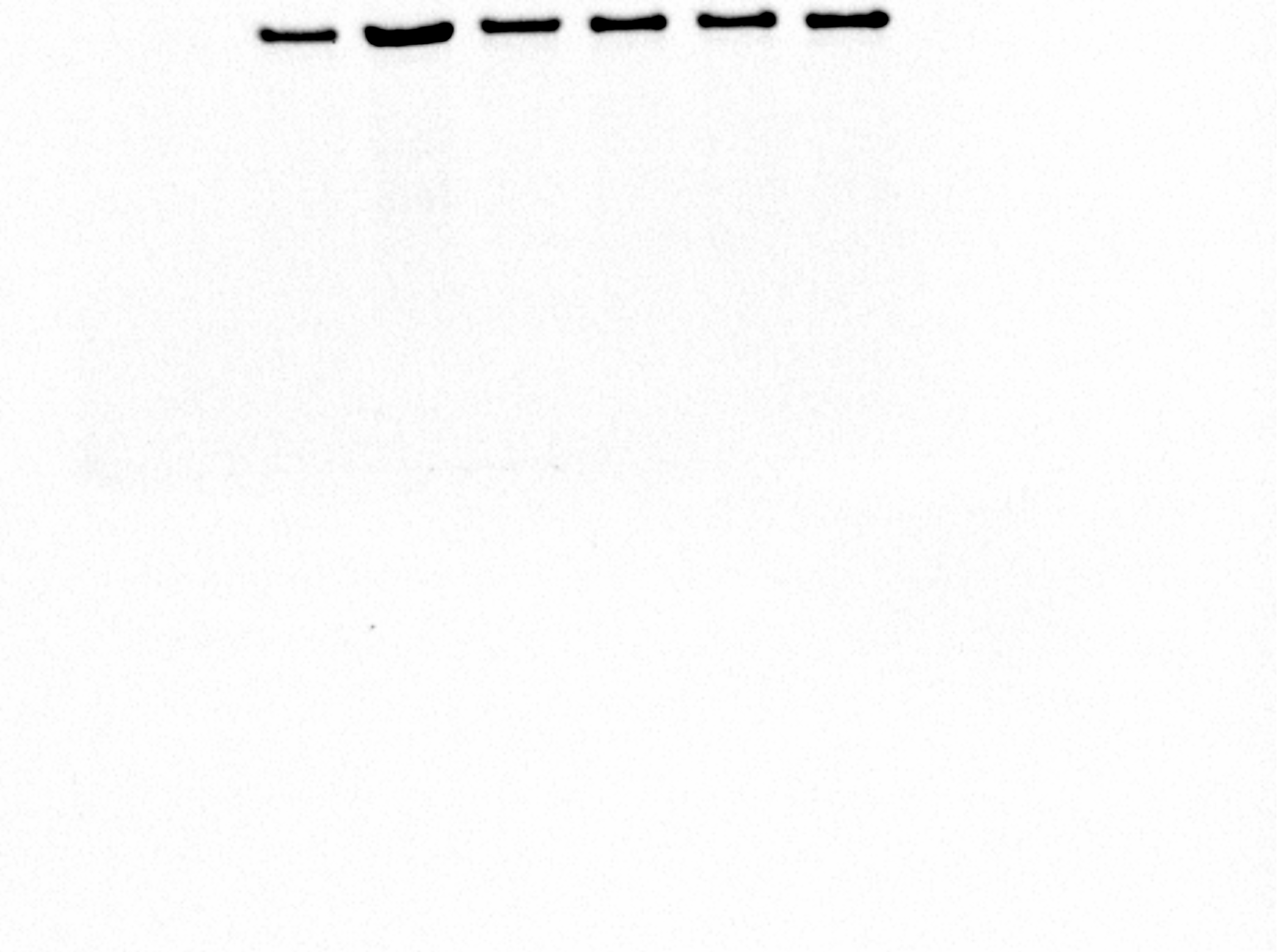

Supplement: Supplementary file 10 [file Image8.TIF]

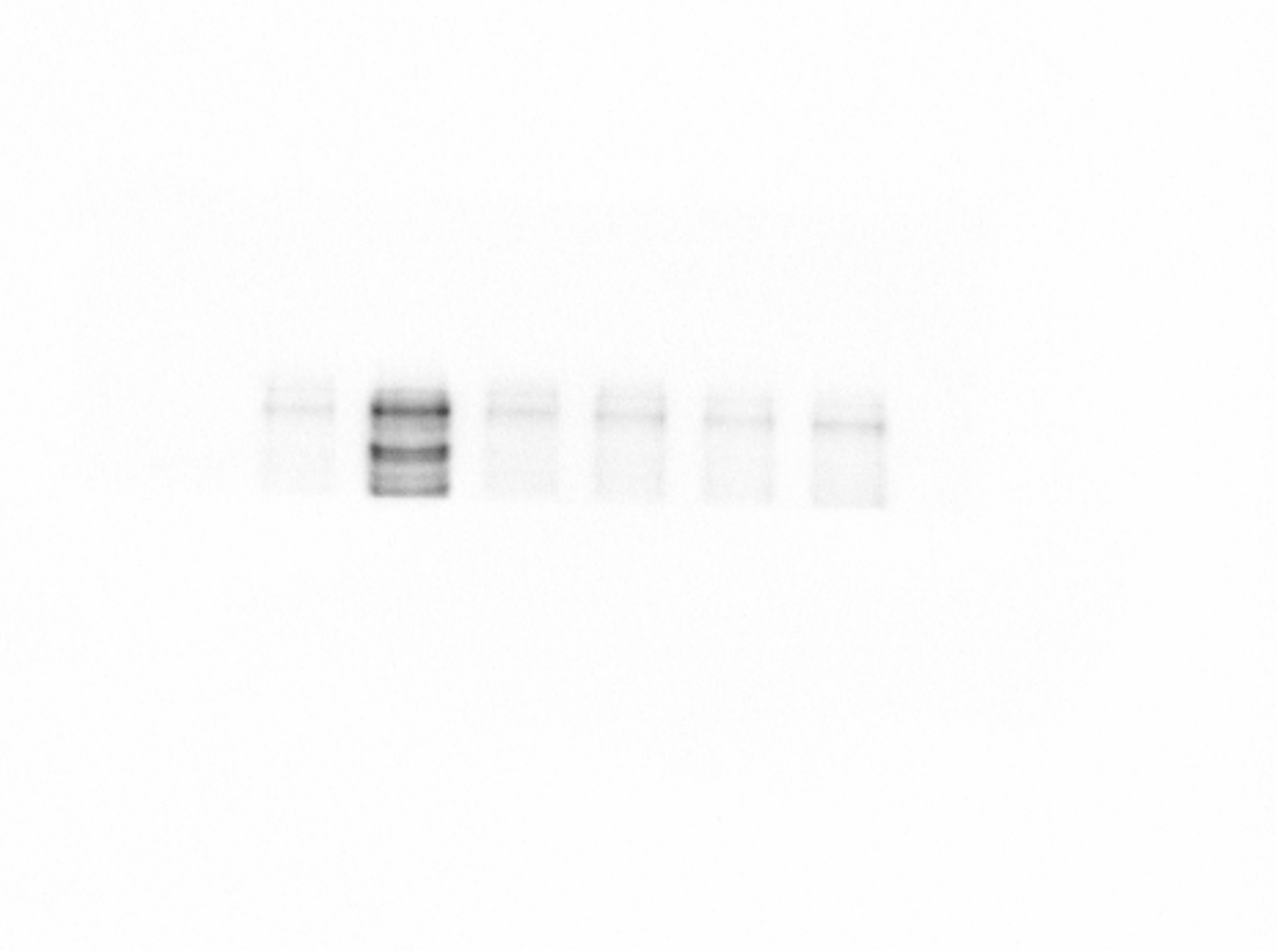

Supplement: Supplementary file 11 [file Image5.TIF]

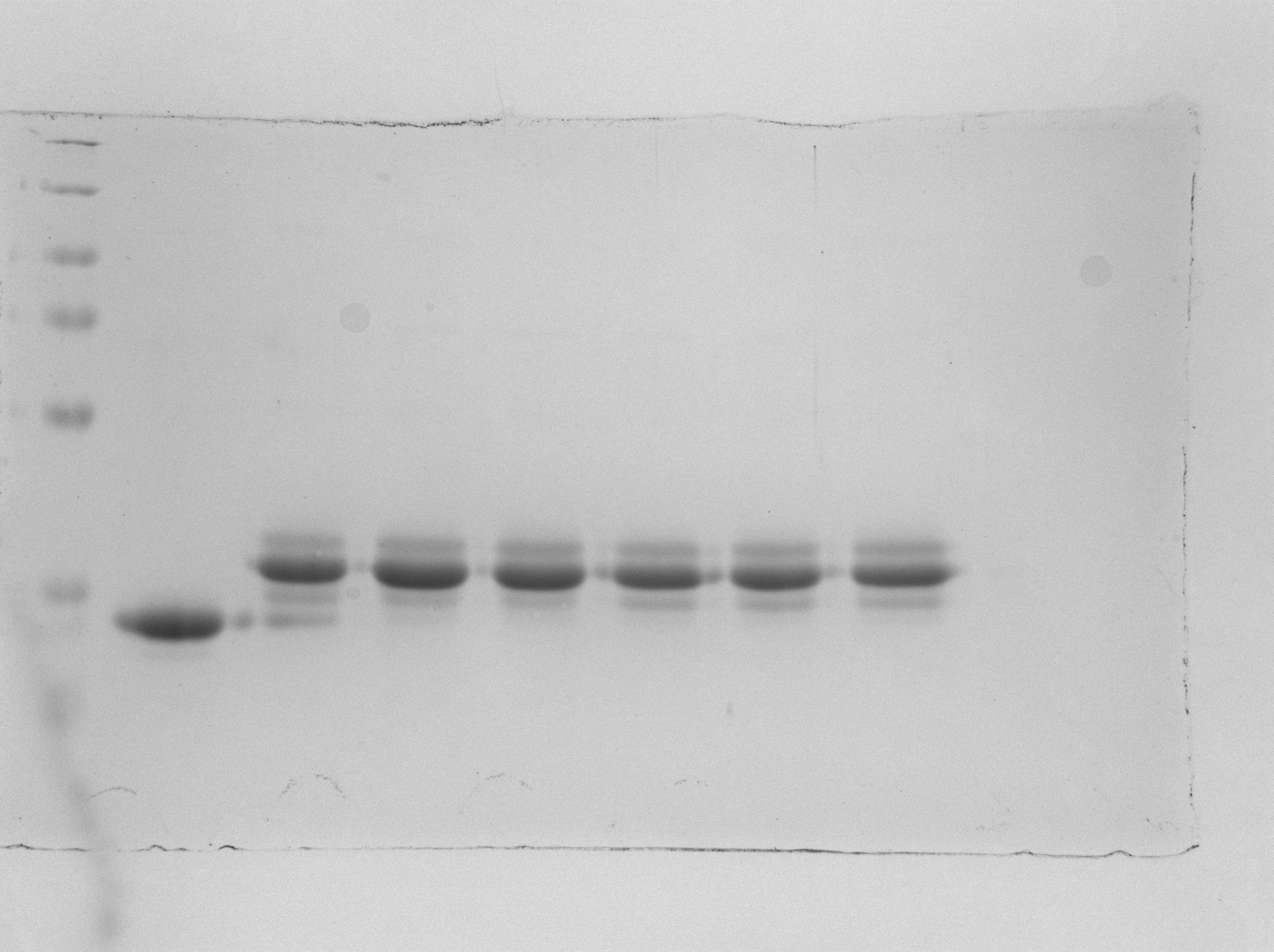

Supplement: Supplementary file 12 [file Image12.TIF]
