# Supplementary material for: Peptibody Based on FGFR1-Binding Peptides From the FGF4 Sequence as a Cancer-Targeting Agent
Source: Front Pharmacol. 2021 Nov 12;12:748936. doi: 10.3389/fphar.2021.748936 (PMC8636100; doi:10.3389/fphar.2021.748936)
Supplement: Supplementary file 13 [file DataSheet1.docx]

Supplementary Material

Supplementary Figure 1.


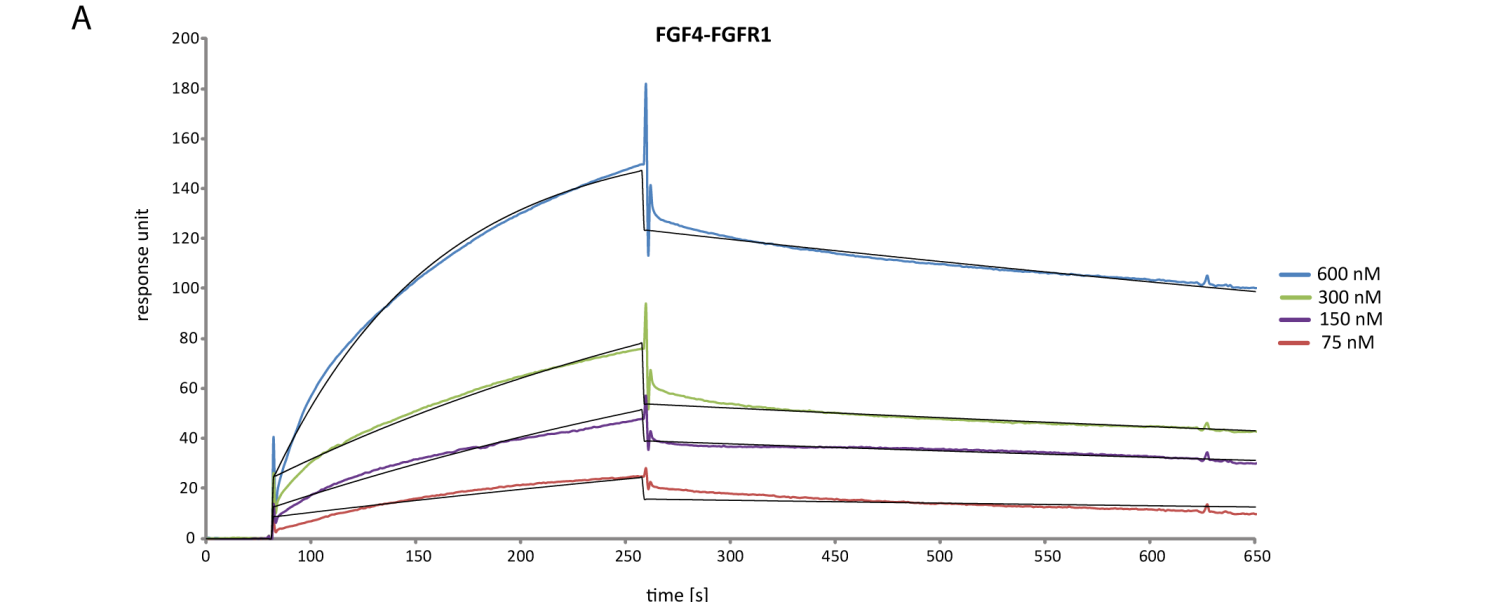


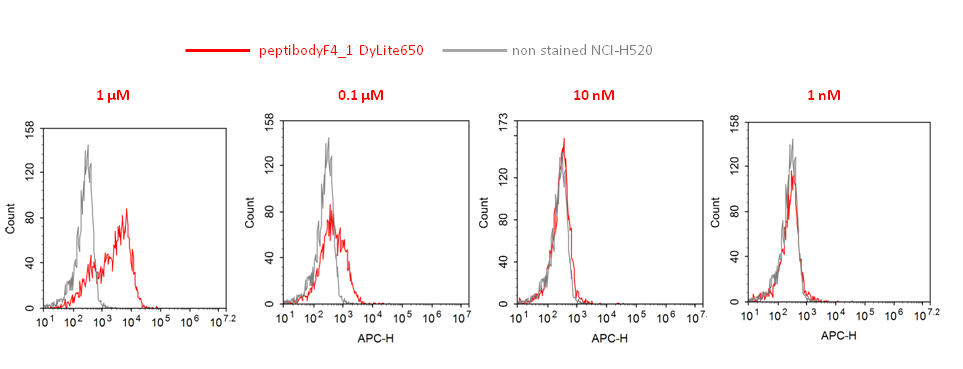

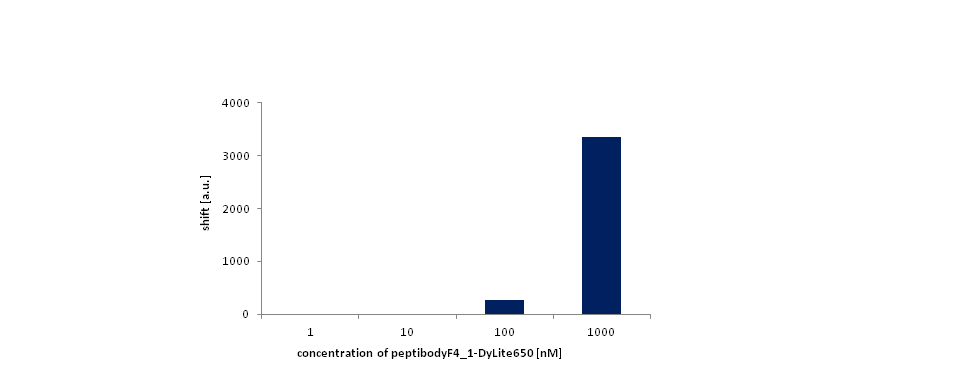


C

B

Supplementary Figure 1. (A) The affinity of FGF4 to FGFR1 was determined by SPR measurements. FGFR1 was immobilized on SPR sensors and incubated with different concentrations of FGF4. Kd values determined for FGF4 was K_D_ =6.57x10^-8^. (B,C) The flow cytometry analysis of peptibodyF4_1 internalization into NCI-H520 cells. Cells were incubated with DyLite650-labeled peptibodyF4_1 (1, 10, 100, 1000 nM) for 30min at 37C, and analyzed by flow cytometry (B). Shift of Mean population values for treated vs. untreated NCI-H520 cells plotted for different peptibodyF4_1 concentrations (C).

Supplementary Figure 2.

A


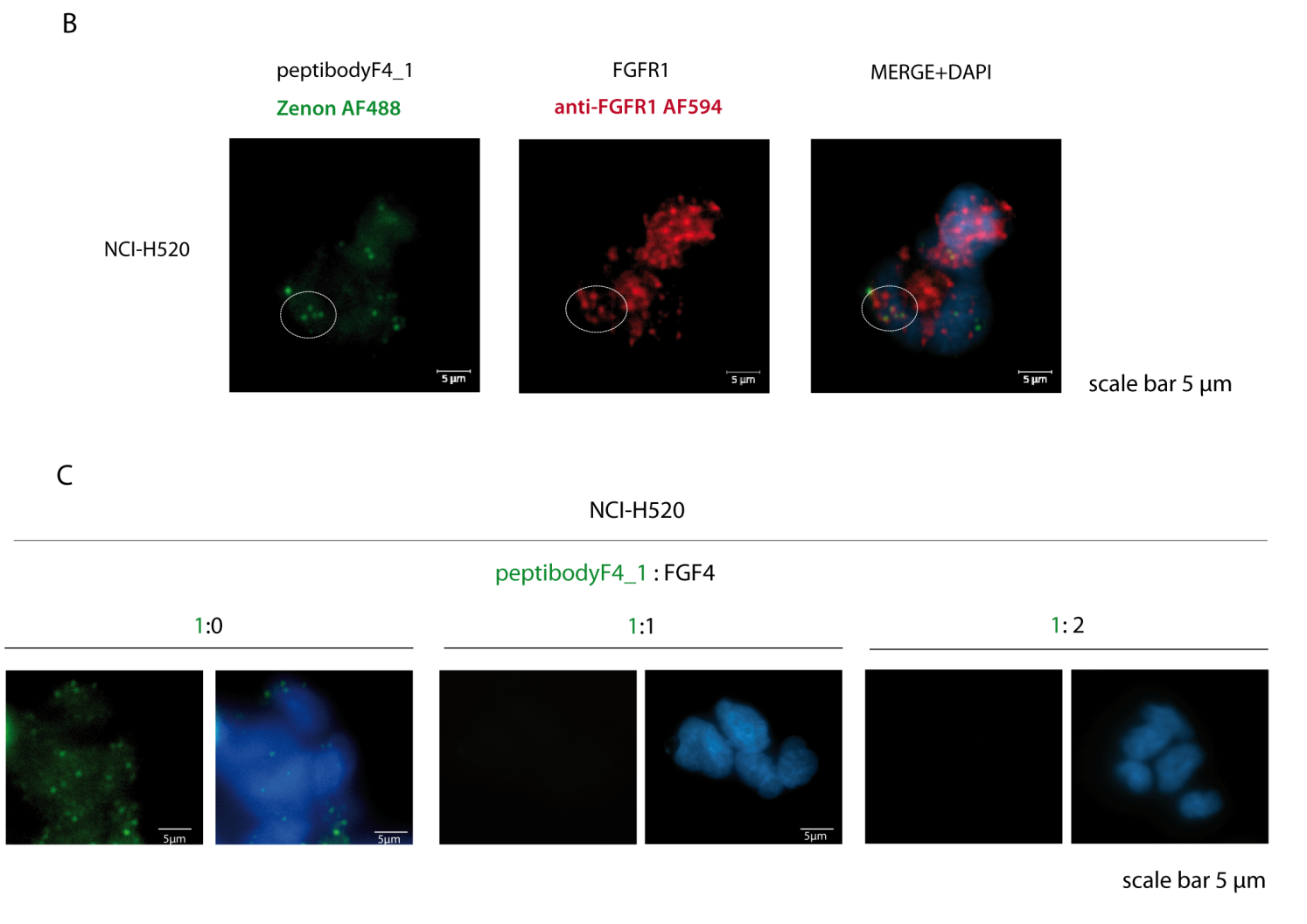


B

Supplementary Figure 2. (A) Colocalization of FGFR1 and peptibodyF4_1. NCI-H520 cells (FGFR1 positive) were incubated with peptibodyF4_1; FGFR1 was detected with anti-FGFR1 antibody (red), peptibody was visualized by Zenon-AF488 reagent, and nuclei were stained with NucBlue reagent. (B) FGF4 can compete-off peptibodyF4_1 binding to NCI-H520 cells. Increasing concentrations of unlabeled FGF4 (natural FGFR1 ligand) and constant peptibodyF4_1 concentrations were added to cells and analyzed by fluorescence microscopy. Relative molar concentrations of peptibodyF4_1 and FGF4 are indicated.

Supplementary Figure 3.


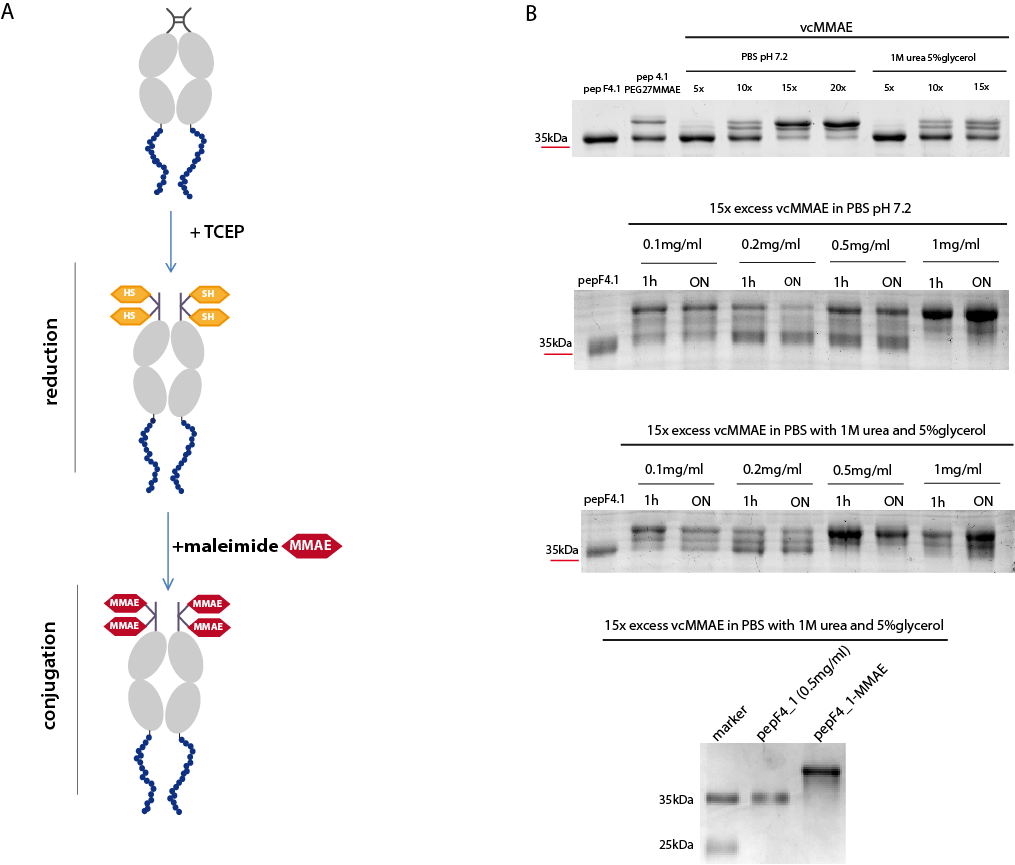


**Supplementary Figure 3.** **Optimization of** s**ite-specific conjugation of cytotoxic drugs to the peptibodyF4_1 (A)** Scheme representing the stages of conjugation with cytotoxic payload via maleimide reaction **(B)** optimization of peptibodyF4_1 conjugation reaction conditions with vcMMAE using maleimide thiol reaction**.**

Supplementary Figure 4.


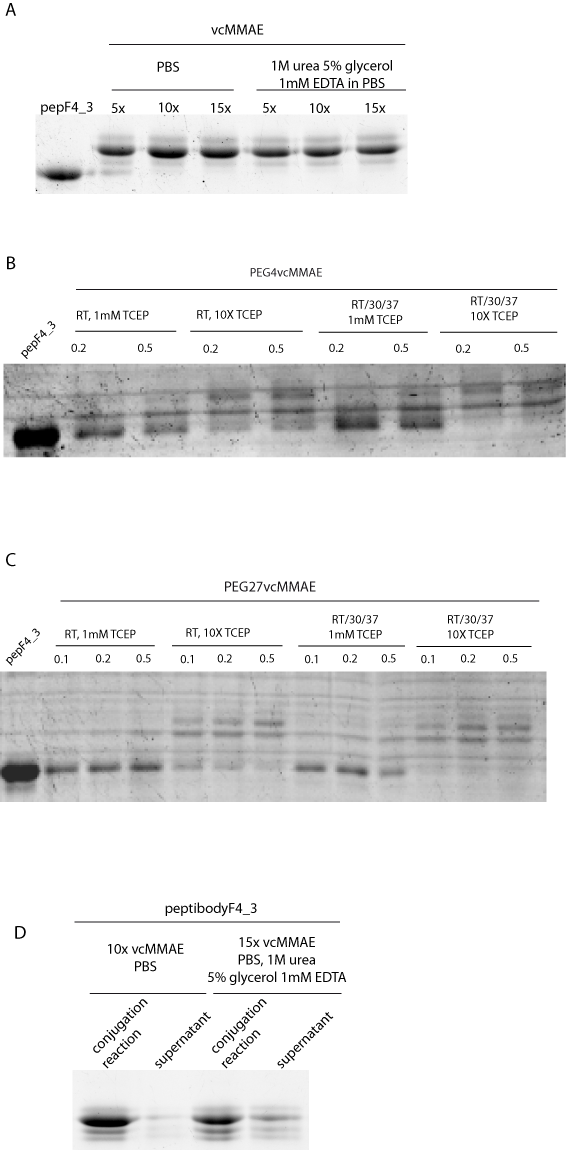


**Supplementary Figure 4.** **Optimization of** s**ite-specific conjugation of cytotoxic drugs to the peptibodyF4_3 (A-C)** Optimization of peptibodyF4_3 conjugation reaction conditions with vcMMAE, PEG27vcMMAE and PEG4vcMMAE using maleimide thiol reaction **(D)** Scaling up conjugation reaction of peptibodyF4_3 with vcMMAE in different buffer reactions (PBS and PBS with 5% glycerol and 1M urea). After incubation with cytotoxic payload conjugates were centrifuged due to visible precipitates.
